# The Development of Cell-Penetrating Peptides for Efficient and Selective In Vivo Expression of mRNA in Spleen Tissue

**DOI:** 10.3390/pharmaceutics15030952

**Published:** 2023-03-15

**Authors:** Ly Porosk, Heleri Heike Härk, Piret Arukuusk, Uku Haljasorg, Pärt Peterson, Kaido Kurrikoff

**Affiliations:** 1 Institute of Technology, University of Tartu, Nooruse 1, 50411 Tartu, Estonia; 2 Vectiopep LLC, Haaviku tee 16, Kambja vald, 61702 Tartu, Estonia; 3 Institute of Biomedicine and Translational Medicine, University of Tartu, 50411 Tartu, Estonia

**Keywords:** cell-penetrating peptides, in vivo transfection, tissue specific expression, mRNA, mRNA delivery, pDNA, freeze-dried nanoparticles, gene delivery

## Abstract

mRNA-based therapeutics are presently one of the nucleic acid-based therapeutics with a high potential for extraordinary success as preventive vaccines. Current applications with mRNA therapeutics rely on lipid nanoparticle (LNP) mediated delivery of nucleic acids. In order to achieve the transition from preventive to therapeutic vaccines, there is a challenge of delivering the mRNA into non-hepatic tissues, especially into lymphoid tissues such as the spleen and lymph nodes. In this work, we characterize new cell-penetrating peptides NF424 and NF436 that exhibit preferential delivery of mRNA into the spleen after a single i.v. injection, without the use of any active targeting mechanisms. We show that between the spleen, liver, and the lungs, >95% of mRNA expression arises in the spleen tissue and the majority of expression occurs in the dendritic cells. The cell-penetrating peptides NF424 and NF436 represent promising candidates for cancer immunotherapeutic applications with tumor antigens.

## 1. Introduction

Transient gene expression technologies are currently a focus of development, as non-viral nucleic acid delivery technologies have succeeded in translating into clinical use. The messenger RNA (mRNA) vaccines particularly achieved success during the pandemic of severe acute respiratory syndrome coronavirus 2 (SARS-CoV-2). This attracted increasing attention in mRNA-based therapeutic possibilities [1]. There are two main ways of inducing exogenous transcript expression: delivering plasmid DNA (pDNA) or delivering mRNA that encodes a protein-of-interest.

Although lipid nanoparticles (LNP-s) offer efficient delivery of nucleic acid in vivo, the challenge is their preferential accumulation in the liver tissue [2,3] and, as a result, the transgene expression is almost exclusively limited to hepatic cells. While this is extremely promising for liver-related diseases, there is a need to achieve nucleic acid delivery to non-hepatic tissues [4], to tackle other clinical modalities that exhibit unmet need, especially cancer. While there are reports with LNP-based methods that succeed in extrahepatic in vivo transfection [5,6,7,8], the underlying accumulation of the LNP into the liver has not been altered. Therefore, non-LNP transfection methods offer a promising way of achieving the use of therapeutic mRNA beyond liver diseases. In this work, we explore the use of cell-penetrating peptides (CPP) as potential carriers for gene vectors with the aim of achieving extrahepatic tissue modulation.

We have previously shown that CPPs PepFect14 (PF14), NickFect55 (NF55), and NickFect71 (NF71) exhibit activity in NA delivery in preclinical models. We previously explored the potential utility of delivering pDNA. For example, PF14 and NF55 mediated pDNA and siRNA delivery into the lungs and lead to effective lung-targeted RNAi [9,10]. We have also explored the potential of delivering small interfering RNA (siRNA) with NF70 and NF71 and achieved efficient gene knock-down in the liver [11]. Moreover, micro-RNA (miRNA) was shown to suppress inflammatory responses in a mouse model of irritant contact dermatitis [12].

However, the delivery of mRNA with CPPs has been less explored [13,14] and we are not aware of studies that compare the potential of transcript expression from pDNA vs. mRNA. Efficient in vivo transfection and subsequent protein expression is one of the limitations in further CPP use for therapeutic purposes. The expression in specific tissues and bypassing tissues generally associated with hepatic side-effects [15,16] would help to increase therapeutic possibilities for nucleic acid-based therapeutics. The aim of this work is to assess the possibility of using CPPs for efficient mRNA delivery in vivo and to design new CPPs with increased efficacy for mRNA delivery into specific tissues in vivo.

## 2. Materials and Methods

### 2.1. Peptide Synthesis

Peptides were synthesized on an automated peptide synthesizer (Biotage, Uppsala, Sweden) using the fluorenylmethyloxycarbonyl (Fmoc) solid phase peptide synthesis strategy with Rink-amide ChemMatrix resin (0.41 mmol/g loading) to obtain C-terminally amidated peptides. The removal of the protective groups (Fmoc) was conducted with 20% piperidine in dimethylformamide (DMF). The reaction was carried out in DMF using HOBT/TBTU (1-hydroxybenzotriazole/2-(1H-benzotriazol-1-yl)-1,1,3,3-tetramethyluronium tetrafluoroborate) for manual or DIC/Oxyma (*N*,*N*′-diisopropylcarbodiimide/ethyl 2-cyano-2-(hydroxyimino)acetate) for machine synthesis as coupling reagents, with DIEA (diisopropylethylamine) as an activator base. Cleavage was performed with trifluoroacetic acid, 2.5% triisopropylsilane, and 2.5% water for 2 h at room temperature. Peptides were purified by reversed-phase high-performance liquid chromatography on a C3 column (Agilent Zorbax 300SB-C3, 5 μm, 250 × 9.4 mm) using a gradient of acetonitrile/water containing 0.1% TFA (trifluoroacetic acid). The molecular weight of the peptides was analyzed by matrix-assisted laser desorption-ionization/time of flight mass spectrometry ((Bruker Daltonics GmbH & Co. KG, Bremen, Germany). The concentration of the peptides was determined based on dilutions of accurately weighed substances and the absorption of tyrosine. Data for peptides after purification are shown in Figure S1.

### 2.2. CPP/NA Complex Mix Preparation

In order to estimate luciferase expression from pDNA and mRNA, we used a plasmid that encodes firefly luciferase under the CMV promoter and compared this with fLuc-encoding mRNA (CleanCap FLuc mRNA that contains 5-methoxyuridine-modified RNA, 1929 nucleotides) (TriLink BioTechnologies, San Diego, CA, USA). For complex preparation, firefly luciferase encoding plasmid (pLuc, 10060 bp, Supplementary Table S1) and mRNA were used. For flow cytometry experiments, enhanced green fluorescent protein-encoding plasmid pEGFP-C1 (4731 bp) (Clontech Laboratories Inc., Montain View, CA, USA) and mRNA mEGFP (CleanCap EGFP mRNA, 996 nucleotides) (TriLink BioTechnologies, San Diego, CA, USA) were used. 

CPP/NA complexes were formed based on the charge ratio (CR) between the nominal positive charge of the peptide and negative charges in the DNA backbone. The CPP/NA complexes were formed by mixing 1 µg of pDNA or mRNA with 1 µL (CR1:1), 2 µL (CR2:1), 3 µL (CR3:1), or 4 µL (CR4:1) of 1 mM NF55, NF430, NF424, or NF436; 0.7 µL (CR1:1), 1.3 µL (CR2:1), 2 µL (CR3:1), or 2.6 µL (CR4:1) of 1 mM NF71; or 0.6 µL (CR1:1) 1.2 µL (CR2:1), 1.8 µL (CR3:1), or 2.4 µL (CR4:1) of 1 mM PF14, diluted in ultrapure water. The complex was formed in 1/10th of the final transfection volume for in vitro experiments. Complexes were incubated for 10–40 min at room temperature before addition to the cells in media or before characterization experiments.

For in vivo complex formation, a 0.5 mg/kg, 1 mg/kg, 1.5 mg/kg, or 2.5 mg/kg NA dose was used. After 20 min incubation at room temperature, 10% glucose was added at an equal volume. Complexes were formed accordingly with the final injection volume of 200–336 µL and contained 5% glucose as an isotonic liquid solution environment. 

For the preparation of freeze-dried complexes (FDC), the complexes were prepared as described for in vivo complex formation and contained 5% glucose as an isotonic liquid solution environment and served as a cryoprotectant for freeze-drying. The injection mix was frozen at −80 °C and freeze-dried. The completely dry mix was kept at +4 °C. Prior to injection, ultrapure water was used to reconstitute the lyophilized mix.

### 2.3. CPP/NA Complex Formation Assessment and CPP/NA Resistance to Enzymatic Digestion by Proteinase K

The complexation of mRNA or pDNA and CPPs was assessed by the Quant-iT™ PicoGreen^®^ (PG) (Thermo Fisher Scientific, Vantaa, Finland) assay in mQ water. Complexes were prepared as described previously, following the addition of diluted PG. For detection, the complexes (10 µL volume), water (90 µL), and PG working dilution (10 µL) were incubated for 5 min and the fluorescence was measured by a fluorimeter (*λ*_ex_ = 492 nm, *λ*_em_ = 535 nm) (SynergyMx, BioTek, Winooski, VT, USA).

pDNA and mRNA condensation by CPPs was analyzed by an ethidium bromide (EtBr) exclusion assay. Complexes were prepared as described for in vitro nanoparticle preparation and after incubation at room temperature, loading dye was added to the complex mix and transferred to the tooth of a 1% agarose gel in 1 x TAE buffer. For imaging purposes, nucleic acid intercalating dye EtBr was added. After electrophoresis, the NA bands were detected.

To assess the protection of mRNA or pDNA from enzymatic degradation, after the addition of PG dilution and the initial measurement to set the starting point, Proteinase K (Thermo Scientific) was added (>20 U per sample), co-incubated with the complexes for 45 min, and the fluorescence was measured to determine the increase of NA accessibility due to the degradation of the CPP.

To assess the resistance of complexes to heparin displacement, complexes were incubated with heparin sodium salt solution at 0, 1, 2, or 3 mg/mL concentration for 30 min at 37 °C. For detection, a complex with heparin (20 µL), water (80 µL), and PG working dilution (10 µL) was incubated for 5 min and the fluorescence was measured by a fluorimeter (*λ*_ex_ = 492 nm, *λ*_em_ = 535 nm) (SynergyMx, BioTek). The results are normalized to the signal of free NA at the same dose (100%) and the background MQ with PG.

### 2.4. Cell Culture Maintenance 

CHO-K1 cells were grown on dishes at 37 °C and 5% CO_2_ in Dulbecco’s Modified Eagle’s Medium (DMEM) (Sigma, Darmstadt, Germany) media supplemented with 0.1 mM non-essential amino acids, 1.0 mM sodium pyruvate, 100 U ml^−1^ penicillin, 100 mg ml^−1^ streptomycin, and 10% fetal bovine serum (FBS). All cell experiments are performed in serum containing media.

### 2.5. In Vitro Transfection Efficacy Assessment: Quantitation of Reporter Levels and the Transfected Cell Population Percentage 

For reporter luminescence (firefly luciferase expressed pDNA or mRNA) assessment in adherent cell culture, 10,000 cells per 96 well-plate well were seeded in 100 µL of media one day before transfection. Shortly prior to transfection, the cell media was replaced with 100 µL of fresh media. The cells were transfected with 0.1 µg of nucleic acid per well and with complexes formed for in vitro transfection (described under Section 2.2). At 24 h post-transfection, the media was aspirated, the cells were washed with 1 × PBS, and 30 µL of lysis buffer (0.1% Triton X100 in 1 × PBS) was added.

Following 20 min incubation at +4 °C for cell lysis, the lysate was mixed and 20 µL was transferred to a black frame white 96-well plate for luminescence measurement after the addition of substrate in a buffer. Luminescence signal was detected with GLOMAX 96 microplate luminometer equipped with GLOMAX 1.9.2 software (Promega Biotech AB, Stockholm, Sweden). RLU values were converted to RLU/mg to normalise against total protein (not reporter protein) in the cell lysate. Total protein reflects the whole amount of cell, therefore by normalisation we reflect the RLU from a specific amount (mg) of protein = cells. For protein detection, the Pierce^TM^ BCA Protein Assay Kit (Thermo Scientific, Vantaa, Finland) was used. Briefly, the absorbance of samples was measured at 562 nm after incubation of samples with a detection mix.

For flow cytometry experiments detecting the transfected cell population (expression from pEGFP-C1 or mEGFP) from adherent cells, 50,000 CHO-K1 cells per well were seeded in 500 µL of serum-containing media on a 24-well plate one day prior to transfection. Shortly before transfection, the media was replaced with 500 µL of fresh media. Cells were transfected with 0.5 µg of pDNA or mRNA per well. At 4 h post-transfection, the media was replaced with 500 µL of fresh serum-containing media. At 24 h post-transfection, the media was removed, the cells were washed with 1 × PBS, and 0.25% trypsin-EDTA was added to detach the cells from the plate. After detachment, 1 × PBS supplemented with 1% FBS was added and the cells were analyzed by flow cytometry to detect the cell population and fluorescence-positive cells. For flow cytometry analysis, an Attune™ NxT Flow Cytometer (Thermo Scientific, Vantaa, Finland) equipped with Attune™ NxT Software 3.2.1 was used. For gating, the forward-scatter (FSC) and side-scatter (SSC) of untreated cells were used. Events with high (clumped cells) or low (debris, complexes, and media components) sizes were excluded. Untreated cells were used to set the GFP+ threshold (~1.5% of untreated were gated as GFP+). For detection, a 488 laser with a 515–545 nm filter was used.

### 2.6. CPP/NA Complex Stability Assessment

The stability of CPP/NA was assessed based on the total reporter levels post-transfection. For this, the CPP/NA nanoparticles were formed as described above at the 1/10th final volume used for transfection for in vitro experiments. The complex mix was kept at +4 °C for 24 h or 1 week. Then, the complex solution was added to CHO-K1 cells in serum containing media. Further details are described above (Section 2.5).

### 2.7. Measurement of CPP/NA Complex Size and Zeta Potential

The hydrodynamic mean diameter and the zeta potential of the nanocomplexes was determined by dynamic light scattering studies using a Zetasizer Nano ZS apparatus (Malvern Panalytical, Malvern, UK). The peptide/NA complexes were prepared in mQ water, as described under Section 2.2, and diluted 10-fold before measurements. All results were based on three measurements from four independent samples. The measurement method and settings were set as follows: material, protein; dispersant, water; and temperature, 22 °C. For particle size determination, the data were converted to “relative by intensity” plots from where the mean hydrodynamic diameter was derived. 

### 2.8. In Vivo Experiments

All animal experiments and procedures were approved by the Estonian Laboratory Animal Ethics Committee (approval no 203, 22 September 2021). For in vivo experiments, male and female 8-week-old BALB/c mice were used. 

The nucleic acid complexes (CPP/mRNA or CPP/pDNA) were prepared as described above, in 336 μL for experiments with 2.5 mg/kg NA and 200 μL per injection for experiments with a lower dose, and injected into the tail vein. The reporter gene expression levels were evaluated after a single injection from the whole tissue homogenate postmortem 24 h or 16 h or with bioluminescence live animal imaging. 

### 2.9. Bioluminescence Live Animal Imaging

Expression of luciferase-encoding mRNA and pDNA was evaluated by in vivo bioluminescence imaging using the IVIS Lumina II (PerkinElmer, Waltham, MA, USA). Mice were injected intraperitoneally (i.p.) with a solution of D-luciferin (PerkinElmer) in DPBS, without Mg^2+^ and Ca^2+^, 150 mg/kg body weight, and sterile-filtered with 0.2 µm syringe filters prior to in vivo use. Mice were anesthetized during imaging using isoflurane, induction 3%, and maintenance 1%. Photon emissions from live animals were quantified 10 min later with an exposure time of 1 min. Regions of interest (ROI) were quantified as average radiance (photons s^−1^ cm^−2^ sr^−1^) represented by the color scale (IVIS Living Image 4.0).

### 2.10. Ex Vivo Reporter Levels in Tissue Homogenates

The tissues were homogenized using the Precellys^®^24-Dual homogenization system (Bertin Technologies, Saint-Quentin en Yveline, France) accompanied with 1× Promega Reporter Lysis buffer or T-PER™ Tissue Protein Extraction Reagent (Thermo Scientific). For the first procedure described in [9], after 15 min incubation with shaking the samples were centrifuged at 10,000× *g*, +4 °C for 3 min, and the supernatant was collected. To the pellet, fresh lysis buffer was added and incubation with shaking was repeated. The supernatants from the same sample were pooled and analyzed. From the cell lysate, luminescence from the expressed firefly luciferase was measured after addition of substrate to the cell lysates. For this, the Luciferase Assay System (Promega, Nacka, Sweden) was used.

To determine the total protein concentrations from the cell lysate, the Pierce™ BCA Protein Assay Kit (Thermo Scientific, Vantaa, Finland) was used. Cell lysate was transferred to a transparent 96-well plate, and 100 µL of solution consisting of component A and component B mixed in a 50:1 ratio was added to each sample. Following 30 min incubation, absorbance was measured at 562 nm. Absorption values were converted to protein concentrations using a standard curve generated by measuring samples with known protein concentrations. Absorption was measured with a Tecan Sunrise microplate absorbance reader (Tecan Austria GmbH, Grödig, Austria). RLU values were converted to RLU/mg to normalise against total protein (not reporter protein) in the cell lysate. Total protein reflects the whole amount of cell, therefore by normalisation we reflect the RLU from a specific amount (mg) of protein = cells.

### 2.11. Spleen Cell Subtype Analysis 

To analyze the cell subtypes that express transfected mLuc, spleens were collected in ice-cold dissociation buffer (1 × PBS without Ca^2+^ and Mg^2+^ supplemented with 2 mM EDTA) 16 h post-transfection with 1.5 mg/kg of mLuc. Following gentle mechanical dissociation by grinding, splenic cells were strained with 100 µm cell strainer and centrifuged at +4 °C, 300× *g*, for 5 min (used throughout). A 2-min incubation with 1 mL of ACK Lysis Buffer was used for red blood cell lysis. Flow cytometry buffer (dissociation buffer supplemented with 0.5% (*w*/*v*) BSA) was added and cells were centrifuged and counted. In total, 20 million cells were incubated with Truestain FcX anti-mouse CD16/32 (BioLegend, San Diego, CA, USA) and stained with antibodies and markers listed in Supplementary Table S2. Cells were washed three times and sorted using Sony MA900 sorter (Sony Europe B.V., Surrey, UK) with a 100 µm sorting chip. Collected cells were centrifuged, lysis buffer was added, and the lysate was analyzed as described under Section 2.5.

### 2.12. Statistical Analysis and Data Visualisation

Statistical analyses (2-way ANOVA) were performed with Statistica software (TIBCO Software Inc., Palo Alto, CA, USA) (2020 Data Science Workbench, version 14). Graph compilation and analysis was performed using GraphPad Prism version 9.5.1 for Windows, GraphPad Software, San Diego, CA, USA.

## 3. Results and Discussion

### 3.1. The Choice of pDNA or mRNA Cargo Affects the Biodistribution of Gene Expression

**Figure 1:**
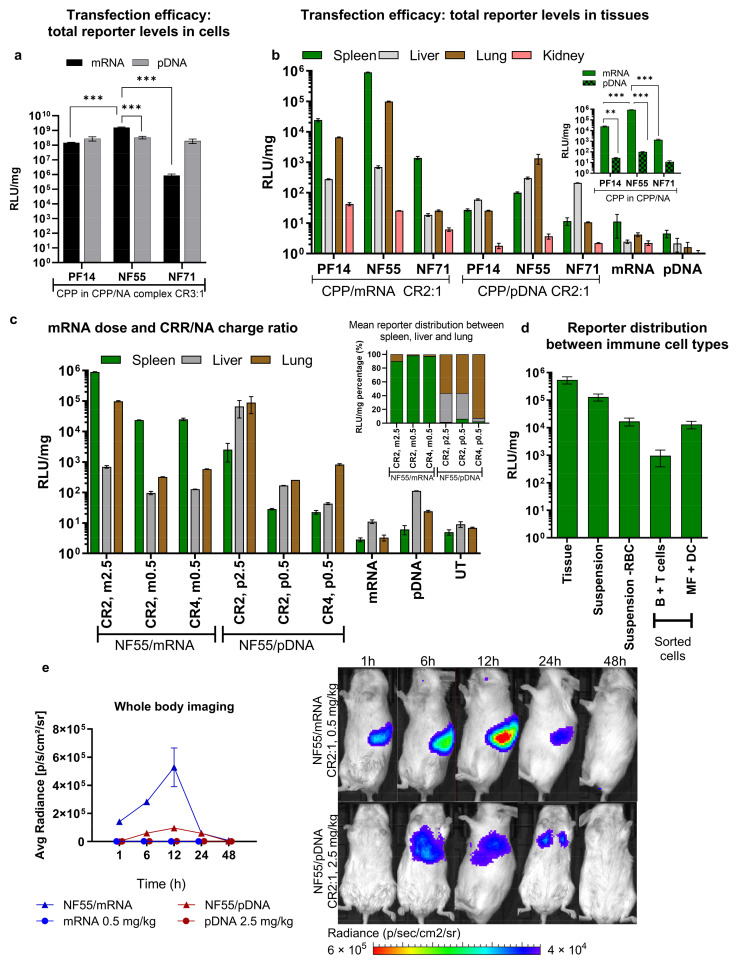
Nanoparticles formed between NF55/mRNA achieve high transfection efficacy in the spleen following systemic administration. (**a**) In vitro transfection of CHO-K1 cells in serum containing conditions. Analyzed 24 h post-transfection. Two-way ANOVA: F(2, 127) = 15.7, *p* = 0.00, Tukey post-hoc comparison: *** = *p* < 0.001. (**b**) CPP/mRNA nanoparticles and c) CPP/pDNA formed at a charge ratio (CR) 2:1 led to different biodistributions of the expressed reporter. pDNA and mRNA dose 2.5 mg/kg. Compared to CPP/pDNA nanoparticles, at the same nucleic acid dose, the CPP/mRNA nanoparticles led to significantly higher expression in the spleen. In (**b**) two-way ANOVA F(3, 16) = 8201.4, *p* = 0.00, Tukey post-hoc comparison: ** = *p* < 0.01, *** = *p* < 0.001. (**c**) mRNA dose reduction in NF55/mRNA nanoparticles results in a significant reduction of expression in the lungs, whereas in the spleen relatively high expression can be achieved even with the lower dose. Comparison of the distribution of reporter between the spleen, liver, and the lungs. NF55/mRNA and NF55/pDNA nanoparticles formed at a charge ratio (CR) 2:1 or 4:1 and with 2.5 mg/kg or 0.5 mg/kg. The distribution of the reporter shown in percentages. (**d**) Reporter levels were detected from spleen cells collected from NF55/mRNA-treated mice. Suspension—RBC stands for cell suspension where red blood cells have been removed. B + T cells—B cells and T cells collected together. MF + DC—macrophages and dendritic cells collected together. For in vivo experiments shown in (**b**–**d**) the tissues were harvested 24 h post-injection. (**e**) The biodistribution and dime-dependency of expression from delivered mRNA and pDNA shows that NF55/mRNA is expressed mainly in the spleen. NF55/mRNA nanoparticles or NF55/pDNA nanoparticles formed at a charge ratio (CR) 2:1. For mRNA-treated groups a 0.5 mg/kg dose was used and for pDNA-treated groups a 2.5 mg/kg NA dose was used. Left panel shows average radiance in the defined spleen region of treated (NF55/NA) and control (mRNA and pDNA) mice over a 1 to 48 h period. Right panels are representative images from whole-body imaging with equalized radiance scales.

To explore the potential of the CPPs PF14, NF55, and NF71 (Table 1) in delivering mRNA type cargo, we compared the delivery and expression of the firefly luciferase (Luc) gene from mRNA or pDNA. As NFs and PFs were used previously for the transfection of cells in the culture, we wanted to compare and confirm if these suit mRNA delivery in vitro. In the case of the pDNA, total reporter levels in CHO-K1 cells were similar for all three CPPs. Interestingly, in the case of mRNA, NF55 achieved more than 10-fold higher RLU/mg values compared to PF14 and NF71 (Figure 1a and Figure S2a). Although both the NF55 and PF14 were able to transfect almost all cells with mRNA (Figure S2b), PF14 did not achieve as high as the total reporter levels. We have previously shown that for the pDNA delivery, both CPPs show similar efficacies and effects on cell viability [17]. Nevertheless, the CPPs may affect other cellular processes, including protein translation or exhibit other unknown undesired side-effects.

We proceeded by investigating how these efficacies achieved in vitro translate in vivo. Surprisingly, the CPPs were significantly more efficient in transfecting the mRNA than pDNA at the same nucleic acid dose (Figure 1b). Based on our previous work with PF14/pDNA, we chose CR2:1 for in vivo complex formation [9]. The best performing CPP was NF55, which induced high expression of luciferase with both mRNA and pDNA. Interestingly, the CPP NF71, which was designed for siRNA delivery [11], also achieved efficient miRNA delivery [12] as well as efficient pDNA delivery [17] (Figure 1a) and negligible reporter expression with mRNA. This could be due to the less optimal complexation–release of NF71/mRNA complexes compared to shorter RNA or larger pDNA. The highest expressions were observed in lung and spleen tissues, but the distribution depended on whether the cargo was RNA or DNA. When using mRNA, the highlighted tissue was spleen, whereas in the case of CPP/pDNA the lungs were highlighted. Nevertheless, NF55 achieved the expression of luciferase that was >700-fold higher with mRNA, compared to what was achieved with pDNA (mRNA in spleen vs. pDNA in lungs). The transfection advantage of mRNA over pDNA may be that the pDNA does not have to reach the nucleus. A similar phenomenon where mRNA offers higher transfection than pDNA was previously shown both in vitro and in vivo with LNP delivery [18].

In the development of cancer vaccines, both used nucleic acids and the delivery method are crucial elements for the achieved delivery efficacy. With the advancement of mRNA-based potential therapeutics, mRNA shows immense potential for this cause. The main delivery method used today are LNP-based. Although the rapid development of LNPs has brought major advancements, the main limitation at this point is the strong expression in the liver and possible hepatic damage post-injection [7]. In the current work we observed high transgene induction in the spleen, and we hypothesized that this could be a beneficial method in immune applications. Particularly for tumor therapy, the targeted delivery and expression of mRNA in the lymphoid organs are considered a promising strategy to improve the efficacy and reduce the side effects of mRNA vaccines [19]. The induction of transgene expression in the spleen is especially interesting with respect to the aim of immunotherapies, as this has the potential for modulating antigen-presenting cells (dendritic cells) with tumor antigens, which in turn can activate cytotoxic T cells [20]. We therefore focused our attention towards the expression of mRNA in the spleen. Luciferase expression in this tissue is magnified in the inset of Figure 1b. 

We next confirmed that lower doses of mRNA can be used to maintain high expression in the spleen and achieve even more favorable spleen-selective activity (Figure S3a). Increasing the charge ratio did not benefit the expression selectivity in the spleen (Figure 1c). When comparing the proportion of the reporter divided between the liver, the lungs, and the spleen (Figure 1c inset), we observed a very strong preference of NF55/mRNA over NF55/pDNA to gain expression especially in the spleen. To gain insight into which types of cells are transfected, we sorted the cells collected from transfected spleens and measured the reporter expression (Figure 1d). We observed that the majority of reporter signal arises from the dendritic cells (Figure S4), further encouraging the potential of CPP-mediated mRNA delivery as a cancer immunotherapeutic application. 

We next gauged the time dynamics of transgene expression and observed that mRNA was already expressed in the spleen at 1 h and achieved its peak at around 12 h (Figure 1e). The expression from pDNA achieved a peak at a later timepoint and, consistently with what we observed from the tissue homogenate analysis (Figure 1b,c), did not achieve as high expression as with mRNA, despite the used mRNA dose being 5 times lower (Figure 1e, ex vivo imaging of harvested tissues at 48 h in Figure S3c). Negative controls (free NA at the same dose) did not result in significant signal compared to NF55/NA complexes (Figure 1e and Figure S3b).

### 3.2. CPPs Form Stable Nanoparticles with mRNA and pDNA

Successful transfection relies on achieving nanoparticle formation with an optimal size and packing of NA. Generally, the optimal range with NF and PF peptides falls between a charge ratio (CR) 2:1 and 3:1 with CPP in excess [9,21], as we also concluded for our complexes in the current study (Figure S5). 

**Figure 2:**
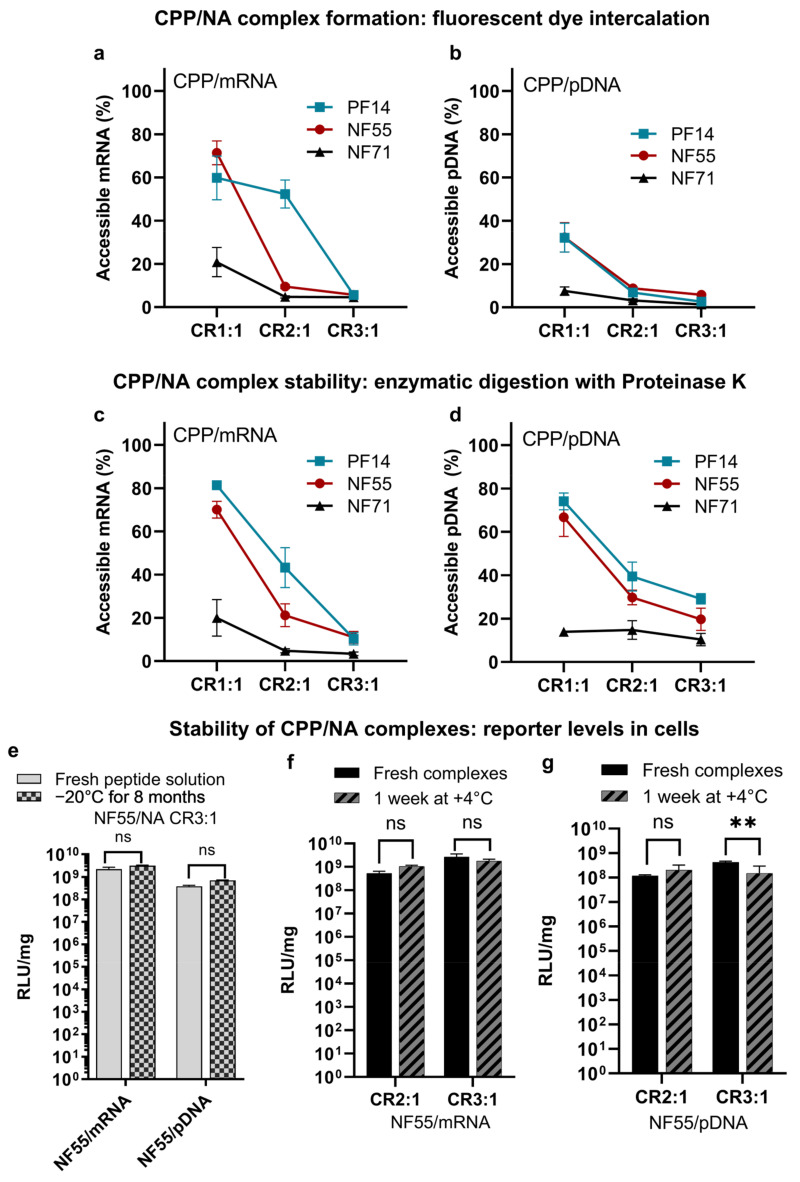
CPPs are able to condense and protect both mRNA and pDNA. Complex formation between CPP and (**a**) mRNA or (**b**) pDNA assessed by accessibility of nucleic acid to fluorescent nucleic acid binding dye after incubation with CPPs. CPP/NA complexes formed at charge ratios (CR) 1:1, 2:1, and 3:1 with CPPs in excess. Free nucleic acid at the same concentration was used as the control (100%). (**c**,**d**) pertain to the stability of pre-formed CPP/NA nanoparticles to enzymatic digestion by proteinase K. The NF55/NA complexes retain their activity even after extended storage. (**e**) Stability of the CPP in water solution. Assessment is based on the reporter levels post-transfection with complexes formed with freshly prepared CPP solution and stored CPP solution. Analyzed 24 h post-transfection. Two-way ANOVA F(1, 35) = 1.2, *p* = 0.27. Tukey post-hoc comparison: ns = not significant. (**f**) CPP/mRNA (two-way ANOVA F(1, 16) = 2.0, *p* = 0.16, and Tukey post-hoc comparison: ns = not significant) and (**g**) CPP/pDNA complexes retain their transfection efficacy even after extended incubation. Analyzed 24 h post-transfection. Two-way ANOVA F(1, 16) = 14.3, *p* = 0.00, Tukey post-hoc comparison: ns = not significant, ** = *p* < 0.01.

To confirm that PF14, NF55, and NF71 associate with both mRNA and pDNA at CR2:1 and CR3:1, we assessed the complex formation between the CPP and the mRNA or pDNA (Figure 2a,b and Figure S5). The complex formation with mRNA resulted in less condensed nanoparticles, as indicated by accessible NA (Figure 2a) compared to only low accessibility of NA in PF14/pDNA complexes at CR1:1 and CR2:1 (Figure 2b). The formation of both CPP/mRNA and CPP/pDNA associations, as indicated by the gel retardation assay, confirmed the optimal CR between 2:1 and 3:1 for all three CPPs and with both pDNA and mRNA. Although the CPP/mRNA nanoparticles were initially less condensed, their stability to degradation was higher compared to CPP/pDNA complexes at CR2:1 and CR3:1. This was indicated by the change of accessible NA ranging between ~5% or no increase in CPP/mRNA (Figure 2c) and 30–40% increase in CPP/pDNA CR2:1 (Figure 2d). This could be due to the differences between the mRNA and pDNA characteristics, such as their size, single-stranded vs. double-stranded nature, and nucleotide modifications. Further studies on the effect of the secondary structure of mRNA vs. pDNA may shed light as to how the type of NA affects the complex formation and the resultant bioactivity. When compared to the CPP/pDNA nanoparticle size distribution, the CPP/mRNA nanoparticle size was less homogenous, especially in the case of NF55 and NF71. In the case of PF14, the size distribution was less varied (Supplementary Table S3). The sizes ranged ~130 to 200 nm for all CPP/NA nanoparticles. When comparing complexes formed between NF55 with pDNA, the complexes formed with PF14 then resulted in nanoparticles with a higher zeta-potential (Table S3).

Stability of the NA, especially the mRNA, is one of the key limitations for its broader therapeutic use [22]. Therefore, we investigated whether the nanoparticles remain stable over an extended incubation time and if the transfection efficacies are preserved. As the first step, we confirmed if the peptide solution in water retains its efficacy over extended storage. For this, we prepared the NF55 solution and tested its transfection efficacy in vitro. An aliquot from the same peptide solution was again tested after eight months (Figure 2e). We did not observe any significant loss of activity after this time period. 

As the next step, we compared NF55/NA nanoparticle aqueous solutions incubated for one week to freshly prepared nanoparticles. The cell culture reporter levels achieved post-transfection did not significantly change over a week for both NF55/mRNA (Figure 2f) and NF55/pDNA (Figure 2g) nanoparticles indicating that the CPPs form stable complexes with mRNA. 

### 3.3. NF55 New Analogs Achieve Further Improved Transgene Expression

As the initial screening indicated clear superiority of NF55 over PF14 and NF71, we screened further variations of NF55 for mRNA delivery. Inspired by our work [23] and previous work on transportan and its deletion analogues [24], we hypothesized that the removal of the first two N-terminal amino acids after the linker, I and N, would not reduce the internalization efficacy of the CPP, but would help to distribute positive charges along the theoretical alpha helix. The deletion of IN in the TP9 did not affect the efficacy compared to TP10, which is one of the parent peptides for NF55. As indicated in [22,23], the NF55 is almost 60% alpha-helical and the C-terminal part of the TP peptide is prone to form an alpha-helical structure. Therefore, we calculated the helical wheel projections to map the cationic amino acid residues. Due to the deletion of two amino acids in the C-terminal region next to the linker amino acid residue, the three amino acid residues with positively charged groups in the peptide would be distributed differently than in NF55 (helical wheel projections in Figure S6). This inspired us to further optimize the complex properties: we replaced the linker amino acid ornithine with 2,4-diaminobutyric acid (Dab) and continued the synthesis from the side chain amino group of the linker amino acid as similarly performed for NF55, resulting in the peptide NF424. To confirm if this replacement of the linker improves efficacy, we also included CPP NF430 where only the deletion is introduced. To further confirm if the distribution of amino groups in the sequence would be beneficial, especially for mRNA delivery, we included an additional modification into the NF424 sequence by changing the positions of KA to AK in the C-terminal part of the peptide, resulting in NF436. (Sequences in Table 1). In the case of pDNA delivery, we have previously shown that the alignment of positive charges in one region along the peptide helical wheel projections is beneficial [23] for in vivo delivery. Due to the modification in the NF430 compared to NF55, the impact of changing KA to AK would be greater than in the case of NF55 and NF54 designed in the abovementioned paper [23]. In addition to the modifications included in the current work, other options such as using D-stereoisomers, the introduction of other charged moieties, peptide stapling, and cyclization, etc., offer immense possibilities for further modifications of peptide-based delivery vectors [25].

**Figure 3:**
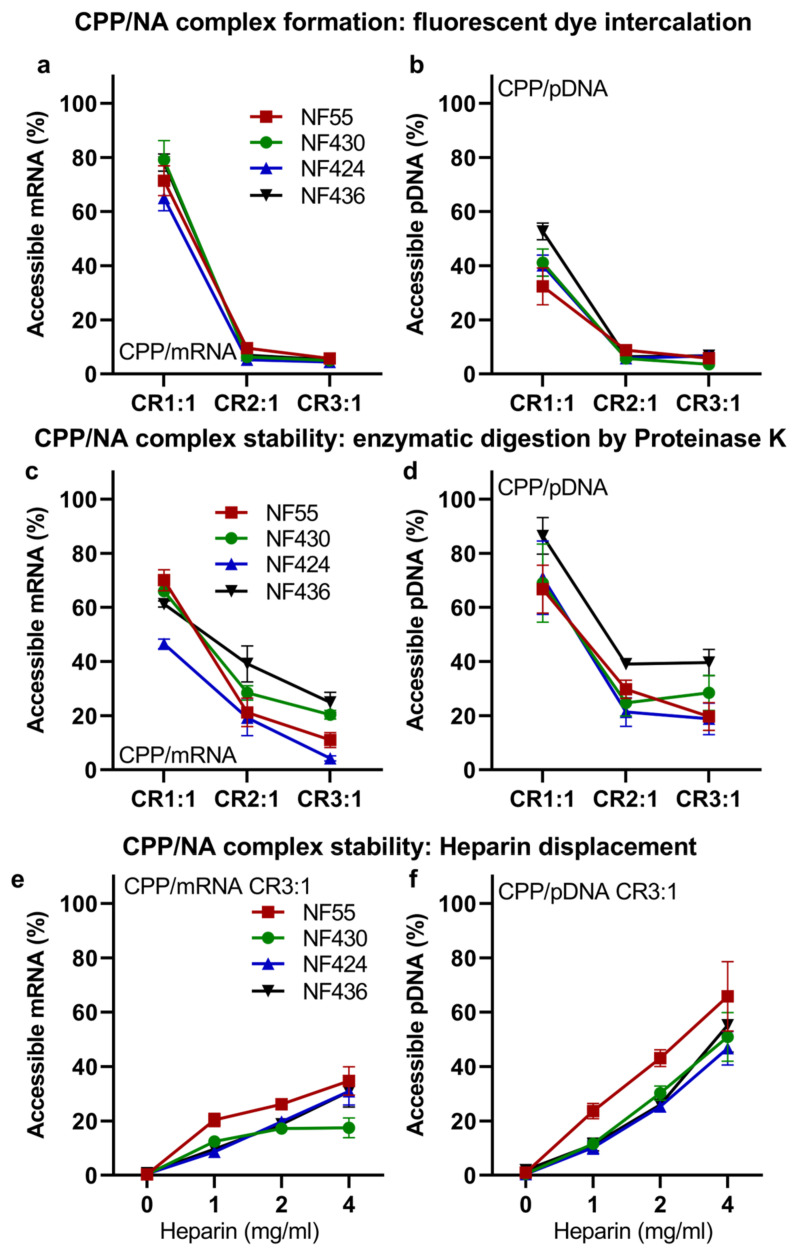
New NF55 analogs form nanoparticles which led to high reporter levels post-transfection both in vitro and in vivo. Complex formation between CPP and (**a**) mRNA or (**b**) pDNA assessed by the accessibility of nucleic acid to fluorescent nucleic acid binding dye after the addition of CPPs. CPP/NA complexes formed at charge ratios (CR) 1:1, 2:1, and 3:1 with CPPs in excess. Free nucleic acid at the same concentration was used as the controls (100%). The stability of pre-formed (**c**) CPP/mRNA and (**d**) CPP/pDNA nanoparticles to enzymatic digestion by proteinase K. The stability of pre-formed (**e**) CPP/mRNA and (**f**) CPP/pDNA nanoparticles to heparin displacement.

**Figure 4:**
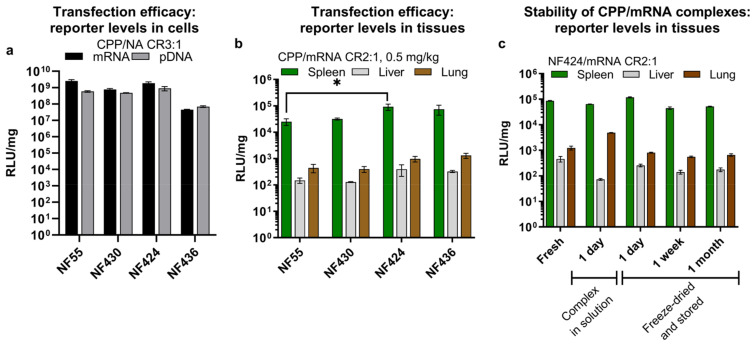
(**a**) Total reporter levels in CHO-K1 after transfection of mRNA or pDNA. A total of 10,000 CHO-K1 cells were seeded one-day prior to transfection on a 96-well plate in 100 µL of serum-containing media. On the transfection day, the media was replaced with fresh media and CPP/NA complexes formed at a charge ratio (CR) of 3:1 were added to the cells. Per each 96-well plate well, 0.1 µg of NA was used. At 24 h post-transfection, cells were washed, lysed, and cell lysate relative luminescence (RLU) was detected after the addition of substrate. RLU is normalized to establish the total protein in the sample (RLU/mg). (**b**) Luciferase reporter levels achieved in vivo. At 16 h post-administration of CPP/mRNA complexes, tissues were harvested and tissue homogenate reporter levels were detected. Two-way ANOVA F(6, 60) = 2.0, *p* = 0.07, Tukey post-hoc comparison: * = *p* < 0.05. (**c**) In vivo comparison of freshly prepared complexes to a complex solution incubated at +4 °C or freeze-dried complexes stored for 1 day, 1 week, or 1 month. Tissues were harvested 16 h post-injection.

Firstly, we confirmed that the new CPPs were able to form stable nanoparticles with both mRNA (Figure 3a) and pDNA (Figure 3b). The nanoparticles with pDNA had an average size of 140 nm and a size of 170 nm with mRNA (Table S2). From the measurement of zeta potentials, we can conclude that the surface charges of the nanoparticles formed with NF55/mRNA nanoparticles and the new analogues had similar zeta-potentials when associated with mRNA, ranging between +33 to +36. Interestingly, when associated with pDNA, the new analog NF430 resulted in complexes with significantly higher zeta-potential when compared to the NF55 and NF436 (Table S3). The modifications did not significantly affect the stability of the CPP/NA to degradation by Proteinase K (Figure 3c,d) or to heparin displacement (Figure 3e,f). As seen for previous in vivo results, the choice of delivered nucleic acid type affects the expressed reporter levels, and for this set, the delivered mRNA was expressed in the spleen cells several folds greater than with the delivered pDNA (Figure 4a and Figure S7). 

As the first indication of delivery efficacy, we confirmed that the new analogs can deliver NA into cells in vitro leading to high reporter expression levels (Figure 4a). Following this, we continued with in vivo evaluation of the CPP/NA complexes. We were mostly interested in determining if even higher mRNA expression can be achieved than what we observed with NF55, and furthermore if the spleen expression can be further elevated. Indeed, all the new NF55 analogs were able to achieve higher spleen mRNA expression at the same mRNA dose (Figure 4b), in the ranking order of NF55 < NF430 < NF436 < NF424 (Figure 4c). NF424 mediated three-fold higher spleen luciferase expression compared to the parent NF55. 

We additionally investigated whether the efficacy of the formed complexes can be preserved in vivo after extended storage as aqueous solutions at +4 °C. We detected a reduction of RLU/mg in the spleen and a slight increase of RLU/mg in the lungs after 24 h storage at +4 °C (Figure 4c). This pushed us to find a more suitable method. Inspired by our previous work on cryo-concentration of complexes [21] and the stability of NF55/pDNA complexes [26], we tested whether the freeze-drying of already formed complexes would help to preserve their activity. Encouragingly, this time the efficacy was retained for one-day complexes and we observed similar luciferase expression in the spleens as with freshly formed mRNA complexes (Figure 4d). When testing complexes stored for one week and one month, we observed some reduction in the total reporter levels. Regardless, the transgene expression achieved with NF424/mRNA was still higher than that obtained with freshly prepared NF55/mRNA (Figure 4b vs. Figure 4c). 

## 4. Conclusions

In the current report, we developed novel cell-penetrating peptides NF424 and NF436 based on NF55 that exhibit significant efficacy in delivering mRNA type cargo, whereas the mRNA expression is largely selective towards the spleen tissue. More specifically, with NF55/mRNA the dendritic cells in the spleen tissue were expressing the mRNA. The significance of this finding is potentially important in terms of cancer immunotherapeutic applications, where tumor antigen-encoding mRNA is used to activate cytotoxic T cells and used as a cancer treatment strategy. 

## Figures and Tables

**Table 1:** Sequences of cell-penetrating peptides used for this work.

CPP	Abbreviation	Sequences
PepFect14 [9,10]	PF14	Stearoyl-AGYLLGKLLOOLAAAALOOLL-NH_2_
NickFect55 [9,10]	NF55	Stearoyl-AGYLLGO ^1^ INLKALAALAKAIL-NH_2_
NickFect71 [11,12]	NF71	Stearoyl-HHYHHGO ^1^ ILLKALKALAKAIL-NH_2_
NickFect430	NF430	Stearoyl-AGYLLGO ^1^ LKALAALAKAIL-NH_2_
NickFect424	NF424	Stearoyl-AGYLLGDab ^2^ LKALAALAKAIL-NH_2_
NickFect436	NF436	Stearoyl-AGYLLGDab ^2^ LKALAALAAKIL-NH_2_

^1^ Synthesis is continued from the side-chain amino group of the ornithine residue. ^2^ Synthesis is continued from the side-chain amino group of 2,4-diaminobutyric acid residue.

## Data Availability

All data are available upon reasonable request from the corresponding author.

## Supplementary Materials

The following supporting information can be downloaded at: https://www.mdpi.com/article/10.3390/pharmaceutics15030952/s1. Supplementary Table S1: Sequence of the reporter pDNA used in this work. Supplementary Table S2: Reagents and antibodies used for cell sorting. Supplementary Table S3: Size distribution of complexes. Figure S1: HPLC and mass spectrometry data of peptides. Figure S2: Total reporter levels and transfected cell population in vitro. Figure S3: In vivo comparison of NF55/mRNA doses and control groups for imaging. Figure S4: Gating strategy for sorting spleen cells. Figure S5: Assessment of CPP/NA complexes with gel migration assay. Figure S6: Helical wheel projections of new analogs compared to NF55. Figure S7: In vivo efficacy of NF55 new analogs for pDNA delivery. Statistics Section: Statistical analysis for figures.

## References

[1] Fang E., Liu X., Li M., Zhang Z., Song L., Zhu B., Wu X., Liu J., Zhao D., Li Y. (2022). Advances in COVID-19 mRNA vaccine development. Signal Transduct. Target. Ther..

[2] Dong Y., Love K.T., Dorkin J.R., Sirirungruang S., Zhang Y., Chen D., Bogorad R.L., Yin H., Chen Y., Vegas A.J. (2014). Lipopeptide nanoparticles for potent and selective siRNA delivery in rodents and nonhuman primates. Proc. Natl. Acad. Sci. USA.

[3] Semple S.C., Akinc A., Chen J., Sandhu A.P., Mui B.L., Cho C.K., Sah D.W., Stebbing D., Crosley E.J., Yaworski E. (2010). Rational design of cationic lipids for siRNA delivery. Nat. Biotechnol..

[4] Loughrey D., Dahlman J.E. (2022). Non-liver mRNA Delivery. Acc. Chem. Res..

[5] Luozhong S., Yuan Z., Sarmiento T., Chen Y., Gu W., McCurdy C., Gao W., Li R., Wilkens S., Jiang S. (2022). Phosphatidylserine Lipid Nanoparticles Promote Systemic RNA Delivery to Secondary Lymphoid Organs. Nano Lett..

[6] Guimaraes P.P., Zhang R., Spektor R., Tan M., Chung A., Billingsley M.M., El-Mayta R., Riley R.S., Wang L., Wilson J.M. (2019). Ionizable lipid nanoparticles encapsulating barcoded mRNA for accelerated in vivo delivery screening. J. Control. Release.

[7] Chen J., Ye Z., Huang C., Qiu M., Song D., Li Y., Xu Q. (2022). Lipid nanoparticle-mediated lymph node-targeting delivery of mRNA cancer vaccine elicits robust CD8^+^ T cell response. Proc. Natl. Acad. Sci. USA.

[8] Dilliard S.A., Cheng Q., Siegwart D.J. (2021). On the mechanism of tissue-specific mRNA delivery by selective organ targeting nanoparticles. Proc. Natl. Acad. Sci. USA.

[9] Kurrikoff K., Veiman K.L., Künnapuu K., Peets E.M., Lehto T., Pärnaste L., Arukuusk P., Langel Ü. (2017). Effective in vivo gene delivery with reduced toxicity, achieved by charge and fatty acid -modified cell penetrating peptide. Sci. Rep..

[10] Kurrikoff K., Freimann K., Veiman K.L., Peets E.M., Piirsoo A., Langel Ü. (2019). Effective lung-targeted RNAi in mice with peptide-based delivery of nucleic acid. Sci. Rep..

[11] Porosk L., Arukuusk P., Põhako K., Kurrikoff K., Kiisholts K., Padari K., Pooga M., Langel Ü. (2019). Enhancement of siRNA transfection by the optimization of fatty acid length and histidine content in the CPP. Biomater. Sci..

[12] Carreras-Badosa G., Maslovskaja J., Periyasamy K., Urgard E., Padari K., Vaher H., Tserel L., Gestin M., Kisand K., Arukuusk P. (2020). NickFect type of cell-penetrating peptides present enhanced efficiency for microRNA-146a delivery into dendritic cells and during skin inflammation. Biomaterials.

[13] van den Brand D., Gorris M.A.J., van Asbeck A.H., Palmen E., Ebisch I., Dolstra H., Hallbrink M., Massuger L., Brock R. (2019). Peptide-mediated delivery of therapeutic mRNA in ovarian cancer. Eur. J. Pharm. Biopharm..

[14] Kim Y., Kim H., Kim E.H., Jang H., Jang Y., Chi S.G., Yang Y., Kim S.H. (2022). The Potential of Cell-Penetrating Peptides for mRNA Delivery to Cancer Cells. Pharmaceutics.

[15] Aldén M., Olofsson Falla F., Yang D., Barghouth M., Luan C., Rasmussen M., De Marinis Y. (2022). Intracellular Reverse Transcription of Pfizer BioNTech COVID-19 mRNA Vaccine BNT162b2 In Vitro in Human Liver Cell Line. Curr. Issues Mol. Biol..

[16] Boettler T., Csernalabics B., Salié H., Luxenburger H., Wischer L., Salimi Alizei E., Zoldan K., Krimmel L., Bronsert P., Schwabenland M. (2022). SARS-CoV-2 vaccination can elicit a CD8 T-cell dominant hepatitis. J. Hepatol..

[17] Porosk L., Nebogatova J., Härk H.H., Vunk B., Arukuusk P., Toots U., Ustav M., Langel Ü., Kurrikoff K. (2022). Predicting Transiently Expressed Protein Yields: Comparison of Transfection Methods in CHO and HEK293. Pharmaceutics.

[18] Perche F., Benvegnu T., Berchel M., Lebegue L., Pichon C., Jaffrès P.A., Midoux P. (2011). Enhancement of dendritic cells transfection in vivo and of vaccination against B16F10 melanoma with mannosylated histidylated lipopolyplexes loaded with tumor antigen messenger RNA. Nanomedicine.

[19] Jiang H., Wang Q., Sun X. (2017). Lymph node targeting strategies to improve vaccination efficacy. J. Control. Release.

[20] Sahin U., Oehm P., Derhovanessian E., Jabulowsky R.A., Vormehr M., Gold M., Maurus D., Schwarck-Kokarakis D., Kuhn A.N., Omokoko T. (2020). An RNA vaccine drives immunity in checkpoint-inhibitor-treated melanoma. Nature.

[21] Freimann K., Arukuusk P., Kurrikoff K., Pärnaste L., Raid R., Piirsoo A., Pooga M., Langel Ü. (2018). Formulation of stable and homogenous cell penetrating peptide NF55 nanoparticles for efficient gene delivery in vivo. Mol. Ther.—Nucleic Acids.

[22] Uddin M.N., Roni M.A. (2021). Challenges of Storage and Stability of mRNA-Based COVID-19 Vaccines. Vaccines.

[23] Freimann K., Arukuusk P., Kurrikoff K., Vasconcelos L.D.F., Veiman K.L., Uusna J., Margus H., Garcia-Sosa A.T., Pooga M., Langel Ü. (2016). Optimization of in vivo DNA delivery with NickFect peptide vectors. J. Control. Release.

[24] Soomets U., Lindgren M., Gallet X., Hällbrink M., Elmquist A., Balaspiri L., Zorko M., Pooga M., Brasseur R., Langel Ü. (2000). Deletion analogues of transportan. Biochim. Biophys. Acta.

[25] Kalafatovic D., Giralt E. (2017). Cell-Penetrating Peptides: Design Strategies beyond Primary Structure and Amphipathicity. Molecules.

[26] Härk H.H., Porosk L., Arukuusk P., Kurrikoff K., Lebl M. (2022). Stability of Cryo-Concentrated Complexes. Proceedings of the 36th European and the 12th International Peptide Symposium.

