# Supplementary material for: The Development of Cell-Penetrating Peptides for Efficient and Selective In Vivo Expression of mRNA in Spleen Tissue
Source: Pharmaceutics. 2023 Mar 15;15(3):952. doi: 10.3390/pharmaceutics15030952 (PMC10055000; doi:10.3390/pharmaceutics15030952)
Supplement: Supplementary file 1 [file pharmaceutics-15-00952-s001.zip › pharmaceutics-2217773-supplementary.pdf]

Supplementary Table S1. The full sequence of the reporter pDNA used for assessment of delivery efficacy.

ACATTACCCTGTTATCCCTAGATGACATTACCCTGTTATCCCAGATGACATTACCCTGTTATCCCTAGATGACATTACCCTGTTATCCCTAGATGACATTTACCCTGTTATCCCTAGATGACATTA
CCCTGTTATCCCAGATGACATTACCCTGTTATCCCTAGATACATTACCCTGTTATCCCAGATGACATACCCTGTTATCCCTAGATGACATTACCCTGTTATCCCAGATGACATTACCCTGTTATCC
CTAGATACATTACCCTGTTATCCCAGATGACATACCCTGTTATCCCTAGATGACATTACCCTGTTATCCCAGATGACATTACCCTGTTATCCCTAGATACATTACCCTGTTATCCCAGATGACATA
CCCTGTTATCCCTAGATGACATTACCCTGTTATCCCAGATGACATTACCCTGTTATCCCTAGATACATTACCCTGTTATCCCAGATGACATACCCTGTTATCCCTAGATGACATTACCCTGTTATCC
CAGATGACATTACCCTGTTATCCCTAGATACATTACCCTGTTATCCCAGATGACATACCCTGTTATCCCTAGATGACATTACCCTGTTATCCCAGATGACATTACCCTGTTATCCCTAGATACATT
ACCCTGTTATCCCAGATGACATACCCTGTTATCCCTAGATGACATTACCCTGTTATCCCAGATAAACTCAATGATGATGATGATGATGGTCGAGACTCAGCGGCCGCGGTGCCAGGGCGTGCC
CTTGGGCTCCCCGGGCGCGACTAGTTCTAGCGAATTCGAGCTCGCCCCGTTACATAACTTACGGTAAATGGCCCGCCTGGCTGACCGCCCAACGACCCCCGCCCATTGACGTCAATAATGACG
TATGTTCCCATAGTAACGCCAATAGGGACTTTCCATTGACGTCAATGGGTGGAGTATTTACGGTAAACTGCCCACTTGGCAGTACATCAAGTGTATCATATGCCAAGTACGCCCCCTATTGACG
TCAATGACGGTAAATGGCCCGCCTGGCATTATGCCCAGTACATGACCTTATGGGACTTTCCTACTTGGCAGTACATCTACGTATTAGTCATCGCTATTACCATGGTGATGCGGTTTTGGCAGTA
CATCAATGGGCGTGGATAGCGGTTTGACTCACGGGGATTTCCAAGTCTCCACCCCATTGACGTCAATGGGAGTTTGTTTTGGCACCAAAATCAACGGGACTTTCCAAAATGTCGTAACAACTC
CGCCCCATTGACGCAAATGGGCGGTAGGCGTGTACGGTGGGAGGTCTATATAAGCAGAGCTCGTTTAGTGAACCGTCAGATCGCCTGGAGACGCCATCCACGCTGTTTTGACCTCCATAGAA
GACACCGGGACCGATCCAGCCTCCGGGGGATCTTGGTGGCGTGAAACTCCCGCACCTCTTCGGCCAGCGCCTTGTAGAAGCGCGTAGGCCTTCTAGCGTACAAGTCCGGACTCAGATCTCGA
GCTCAAGCTTCGGCGACGTCGCCACGTGATGGGAGTCAAAGTTCTGTTTGCCCTGATCTGCATCGCTGTGGCCGAGGCCAAGCCCACCGAGAACAACGAAGACTTCAACATCGTGGCCGTGG
CCAGCAACTTCGCGACCACGGATCTCGATGCTGACCGCGGGAAGTTGCCCGGCAAGAAGCTGCCGCTGGAGGTGCTCAAAGAGATGGAAGCCAATGCCCGGAAAGCTGGCTGCACCAGGG
GCTGTCTGATCTGCCTGTCCCACATCAAGTGCACGCCCAAGATGAAGAAGTTCATCCCAGGACGCTGCCACACCTACGAAGGCGACAAAGAGTCCGCACAGGGCGGCATAGGCGAGGCGAT
CGTCGACATTCCTGAGATTCCTGGGTTCAAGGACTTGGAGCCCATGGAGCAGTTCATCGCACAGGTCGATCTGTGTGTGGACTGCACAACTGGCTGCCTCAAAGGGCTTGCCAACGTGCAGT
GTTCTGACCTGCTCAAGAAGTGGCTGCCGCAACGCTGTGCGACCTTTGCCAGCAAGATCCAGGGCCAGGTGGACAAGATCAAGGGGGCCGGTGGTGACTAAACCGGTCGACAAGGTGAGT
TTGGGGACCCTTGATTGTTCTTTCTTTTTCGCTATTGTAAAATTCATGTTATATGGAGGGGGCAAAGTTTTCAGGGTGTTGTTTAGAATGGGAAGATGTCCCTTGTATCACCATGGACCCTCAT
GATAATTTTGTTTCTTTCACTTTCTACTCTGTTGACAACCATTGTCTCCTCTTATTTTCTTTTCATTTTCTGTAACTTTTTCGTTAAACTTTAGCTTGCATTTGTAACGAATTTTTAAATTCACTTTTGT
TTATTTGTCAGATTGTAAGTACTTTCTCTAATCACTTTTTTTTCAAGGCAATCAGGGTATATTATATTGTACTTCAGCACAGTTTTAGAGAACAATTGTTATAATTAAATGATAAGGTAGAATATT
TCTGCATATAAATTCTGGCTGGCGTGGAAATATTCTTATTGGTAGAAACAACTACACCCTGGTCATCATCCTGCCTTTCTCTTTATGGTTACAATGATATACACTGTTTGAGATGAGGATAAAAT
ACTCTGAGTCCAAACCGGGCCCCTCTGCTAACCATGTTCATGCCTTCTTCTTTTTCCTACAGGCTCGACGGAGATGGGGGAGGCTAACTGAAACACGGAAGGAGACAATACCGGAAGGAACC
CGCGCTATGACGGCAATAAAAAGACAGAATAAAACGCACGGGTGTTGGGTCGTTTGTTCATAAACGCGGGGTTCGGTCCCAGGGCTGGCACTCTGTCGATACCCCACCGAGACCCCATTGG
GGCCAATACGCCCGCGTTTCTTCCTTTTCCCCACCCCACCCCCCAAGTTCGGGTGAAGGCCCAGGGCTCGCAGCCAACGTCGGGGCGGCAGGCCCTGCCATAGCCACTGGCCCCGTGGGTTA
GGGACGGGGTCCCCATGGGGAATGGTTTATGGTTCGTGGGGGTTATTATTTTGGGCGTTGCGTGGGGTCTGGGTATACCCTCGACCTGCAGGGTACCAGATCTGCTAGTTCTAGCGAATTCG
AGCTCGCCCCGTTACATAACTTACGGTAAATGGCCCGCCTGGCTGACCGCCCAACGACCCCCGCCCATTGACGTCAATAATGACGTATGTTCCCATAGTAACGCCAATAGGGACTTTCCATTGA
CGTCAATGGGTGGAGTATTTACGGTAAACTGCCCACTTGGCAGTACATCAAGTGTATCATATGCCAAGTACGCCCCCTATTGACGTCAATGACGGTAAATGGCCCGCCTGGCATTATGCCCAG
TACATGACCTTATGGGACTTTCCTACTTGGCAGTACATCTACGTATTAGTCATCGCTATTACCATGGTGATGCGGTTTTGGCAGTACATCAATGGGCGTGGATAGCGGTTTGACTCACGGGGA
TTTCCAAGTCTCCACCCCATTGACGTCAATGGGAGTTTGTTTTGGCACCAAAATCAACGGGACTTTCCAAAATGTCGTAACAACTCCGCCCCATTGACGCAAATGGGCGGTAGGCGTGTACGG
TGGGAGGTCTATATAAGCAGAGCTCGTTTAGTGAACCGTCAGATCGCCTGGAGACGCCATCCACGCTGTTTTGACCTCCATAGAAGACACCGGGACCGATCCAGCCTCCGGGGGATCTTGGT
GGCGTGAAACTCCCGCACCTCTTCGGCCAGCGCCTTGTAGAAGCGCGTAGGCCTTCTAGCGTACAAGTCCGGACTCAGATCTCGAGCTCAAGCTTCGAAATGGAAGATGCCAAAAACATTAA
GAAGGGCCCAGCGCCATTCTACCCACTCGAAGACGGGACCGCCGGCGAGCAGCTGCACAAAGCCATGAAGCGCTACGCCCTGGTGCCCGGCACCATCGCCTTTACCGACGCACATATCGAG
GTGGACATTACCTACGCCGAGTACTTCGAGATGAGCGTTCGGCTGGCAGAAGCTATGAAGCGCTATGGGCTGAATACAAACCATCGGATCGTGGTGTGCAGCGAGAATAGCTTGCAGTTCTT
CATGCCCGTGTTGGGTGCCCTGTTCATCGGTGTGGCTGTGGCCCCAGCTAACGACATCTACAACGAGCGCGAGCTGCTGAACAGCATGGGCATCAGCCAGCCCACCGTCGTATTCGTGAGCA
AGAAAGGGCTGCAAAAGATCCTCAACGTGCAAAAGAAGCTACCGATCATACAAAAGATCATCATCATGGATAGCAAGACCGACTACCAGGGCTTCCAAAGCATGTACACCTTCGTGACTTCC

CATTTGCCACCCGGCTTCAACGAGTACGACTTCGTGCCCGAGAGCTTCGACCGGGACAAAACCATCGCCCTGATCATGAACAGTAGTGGCAGTACCGGATTGCCCAAGGGCGTAGCCCTACC
GCACCGCACCGCTTGTGTCCGATTCAGTCATGCCCGCGACCCCATCTTCGGCAACCAGATCATCCCCGACACCGCTATCCTCAGCGTGGTGCCATTTCACCACGGCTTCGGCATGTTCACCACG
CTGGGCTACTTGATCTGCGGCTTTCGGGTCGTGCTCATGTACCGCTTCGAGGAGGAGCTATTCTTGCGCAGCTTGCAAGACTATAAGATTCAATCTGCCCTGCTGGTGCCCACACTATTTAGCT
TCTTCGCTAAGAGCACTCTCATCGACAAGTACGACCTAAGCAACTTGCACGAGATCGCCAGCGGCGGGGCGCCGCTCAGCAAGGAGGTAGGTGAGGCCGTGGCCAAACGCTTCCACCTACC
AGGCATCCGCCAGGGCTACGGCCTGACAGAAACAACCAGCGCCATTCTGATCACCCCCGAAGGGGACGACAAGCCTGGCGCAGTAGGCAAGGTGGTGCCCTTCTTCGAGGCTAAGGTGGT
GGACTTGGACACCGGTAAGACACTGGGTGTGAACCAGCGCGGCGAGCTGTGCGTCCGTGGCCCCATGATCATGAGCGGCTACGTTAACAACCCCGAGGCTACAAACGCTCTCATCGACAAG
GACGGCTGGCTGCACAGCGGCGACATCGCCTACTGGGACGAGGACGAGCACTTCTTCATCGTGGACCGGCTGAAGAGCCTGATCAAATACAAGGGCTACCAGGTAGCCCCAGCCGAACTGG
AGAGCATCCTGCTGCAACACCCCAACATCTTCGACGCCGGGGTCGCCGGCCTGCCCGACGACGATGCCGGCGAGCTGCCCGCCGCAGTCGTCGTGCTGGAACACGGTAAAACCATGACCGA
GAAGGAGATCGTGGACTATGTGGCCAGCCAGGTTACAACCGCCAAGAAGCTGCGCGGTGGTGTTGTGTTCGTGGACGAGGTGCCTAAAGGACTGACCGGCAAGTTGGACGCCCGCAAGAT
CCGCGAGATTCTCATTAAGGCCAAGAAGGGCGGCAAGATCGCCGTGTAACCGCGGTACCAGGTCGACAAGGTGAGTTTGGGGACCCTTGATTGTTCTTTCTTTTTCGCTATTGTAAAATTCAT
GTTATATGGAGGGGGCAAAGTTTTCAGGGTGTTGTTTAGAATGGGAAGATGTCCCTTGTATCACCATGGACCCTCATGATAATTTTGTTTCTTTCACTTTCTACTCTGTTGACAACCATTGTCTC
CTCTTATTTTCTTTTCATTTTCTGTAACTTTTTCGTTAAACTTTAGCTTGCATTTGTAACGAATTTTTAAATTCACTTTTGTTTATTTGTCAGATTGTAAGTACTTTCTCTAATCACTTTTTTTTCAAGG
CAATCAGGGTATATTATATTGTACTTCAGCACAGTTTTAGAGAACAATTGTTATAATTAAATGATAAGGTAGAATATTTCTGCATATAAATTCTGGCTGGCGTGGAAATATTCTTATTGGTAGA
AACAACTACACCCTGGTCATCATCCTGCCTTTCTCTTTATGGTTACAATGATATACACTGTTTGAGATGAGGATAAAATACTCTGAGTCCAAACCGGGCCCCTCTGCTAACCATGTTCATGCCTT
CTTCTTTTTCCTACAGGCTCGACGGAGATGGGGGAGGCTAACTGAAACACGGAAGGAGACAATACCGGAAGGAACCCGCGCTATGACGGCAATAAAAAGACAGAATAAAACGCACGGGTG
TTGGGTCGTTTGTTCATAAACGCGGGGTTCGGTCCCAGGGCTGGCACTCTGTCGATACCCCACCGAGACCCCATTGGGGCCAATACGCCCGCGTTTCTTCCTTTTCCCCACCCCACCCCCCAAG
TTCGGGTGAAGGCCCAGGGCTCGCAGCCAACGTCGGGGCGGCAGGCCCTGCCATAGCCACTGGCCCCGTGGGTTAGGGACGGGGTCCCCATGGGGAATGGTTTATGGTTCGTGGGGGTTA
TTATTTTGGGCGTTGCGTGGGGTCTGGGTATACCCTCGACCTGCAGGGTACCAGATCTGCTAGCTAGTCTTTCCGATCGATGGAAGGATCCGTCGGAGCTCTACCTTGCGGCCGCGACATGAT
AAGATACATTGATGAGTTTGGACAAACCACAACTAGAATGCAGTGAAAAAAATGCTTTATTTGTGAAATTTGTGATGCTATTGCTTTATTTGTGAAATTTGTGATGCTATTGCTTTATTTGTAAC
CATTATAAGCTGCAATAAACAAGTTAACAACAACAATTGCATTCATTTTATGTTTCAGGTTCAGGGGGAGGTGTGGGAGGTTTTTTAAAGCAAGTAAAACCTCTACAAATGTGGTAGATCATT
TATCTCTAGAGTCGACCCATGGGGGCCCGCCCCAACTGGGGTAACCTTTGAGTTCTCTCAGTTGGGGGTAATCAGCATCATGATGTGGTACCACATCATGATGCTGATTATAAGAATGCGGCC
GCCACACTCTAGTGGATCTCGAGTTAATAATTCAGAAGAACTCGTCAAGAAGGCGATAGAAGGCGATGCGCTGCGAATCGGGAGCGGCGATACCGTAAAGCACGAGGAAGCGGTCAGCCC
ATTCGCCGCCAAGCTCTTCAGCAATATCACGGGTAGCCAACGCTATGTCCTGATAGCGGTCCGCCACACCCAGCCGGCCACAGTCGATGAATCCAGAAAAGCGGCCATTTTCCACCATGATAT
TCGGCAAGCAGGCATCGCCATGGGTCACGACGAGATCCTCGCCGTCGGGCATGCTCGCCTTGAGCCTGGCGAACAGTTCGGCTGGCGCGAGCCCCTGATGCTCTTCGTCCAGATCATCCTGA
TCGACAAGACCGGCTTCCATCCGAGTACGTGCTCGCTCGATGCGATGTTTCGCTTGGTGGTCGAATGGGCAGGTAGCCGGATCAAGCGTATGCAGCCGCCGCATTGCATCAGCCATGATGGA
TACTTTCTCGGCAGGAGCAAGGTGTAGATGACATGGAGATCCTGCCCCGGCACTTCGCCCAATAGCAGCCAGTCCCTTCCCGCTTCAGTGACAACGTCGAGCACAGCTGCGCAAGGAACGCC
CGTCGTGGCCAGCCACGATAGCCGCGCTGCCTCGTCTTGCAGTTCATTCAGGGCACCGGACAGGTCGGTCTTGACAAAAAGAACCGGGCGCCCCTGCGCTGACAGCCGGAACACGGCGGCA
TCAGAGCAGCCGATTGTCTGTTGTGCCCAGTCATAGCCGAATAGCCTCTCCACCCAAGCGGCCGGAGAACCTGCGTGCAATCCATCTTGTTCAATCATGCGAAACGATCCTCATCCTGTCTCTT
GATCAGAGCTTGATCCCCTGCGCCATCAGATCCTTGGCGGCGAGAAAGCCATCCAGTTTACTTTGCAGGGCTTCCCAACCTTACCAGAGGGCGCCCCAGCTGGCAATTCCGGTTCGCTTGCTG
TCCATAAAACCGCCCAGTCTAGCTATCGCCATGTAAGCCCACTGCAAGCTACCTGCTTTCTCTTTGCGCTTGCGTTTTCCCTTGTCCAGATAGCCCAGTAGCTGACATTCATCCGGGGTCAGCAC
CGTTTCTGCGGACTGGCTTTCTACGTGCTCGAGGGGGGCCAAACGGTCTCCAGCTTGGCTGTTTTGGCGGATGAGAGAAGATTTTCAGCCTGATACAGATTAAATCAGAACGCAGAAGCGGT
CTGATAAAACAGAATTTGCCTGGCGGCAGTAGCGCGGTGGTCCCACCTGACCCCATGCCGAACTCAGAAGTGAAACGCCGTAGCGCCGATGGTAGTGTGGGGTCTCCCCATGCGAGAGTAG
GGAACTGCCAGGCATCAAATAAAACGAAAGGCTCAGTCGAAAGACTGGGCCTTTCGTTTTATCTGTTGTTTGTCGGTGAACGCTCTCCTGAGTAGGACAAATCCGCCGGGAGCGGATTTGAA
CGTTGCGAAGCAACGGCCCGGAGGGTGGCGGGCAGGACGCCCGCCATAAACTGCCAGGCATCAAATTAAGCAGAAGGCCATCCTGACGGATGGCCTTTTTGCGTTTCTACAAACTCTTTTGT
TTATTTTTCTAAATACATTCAAATATGTATCCGCTCATGACCAAAATCCCTTAACGTGAGTTTTCGTTCCACTGAGCGTCAGACCCCGTAGAAAAGATCAAAGGATCTTCTTGAGATCCTTTTTTT
CTGCGCGTAATCTGCTGCTTGCAAACAAAAAAACCACCGCTACCAGCGGTGGTTTGTTTGCCGGATCAAGAGCTACCAACTCTTTTTCCGAAGGTAACTGGCTTCAGCAGAGCGCAGATACCA
AATACTGTCCTTCTAGTGTAGCCGTAGTTAGGCCACCACTTCAAGAACTCTGTAGCACCGCCTACATACCTCGCTCTGCTAATCCTGTTACCAGTGGCTGCTGCCAGTGGCGATAAGTCGTGTC
TTACCGGGTTGGACTCAAGACGATAGTTACCGGATAAGGCGCAGCGGTCGGGCTGAACGGGGGGTTCGTGCACACAGCCCAGCTTGGAGCGAACGACCTACACCGAACTGAGATACCTACA

GCGTGAGCTATGAGAAAGCGCCACGCTTCCCGAAGGGAGAAAGGCGGACAGGTATCCGGTAAGCGGCAGGGTCGGAACAGGAGAGCGCACGAGGGAGCTTCCAGGGGGAAACGCCTGG
TATCTTTATAGTCCTGTCGGGTTTCGCCACCTCTGACTTGAGCGTCGATTTTTGTGATGCTCGTCAGGGGGGCGGAGCCTATGGAAAAACGCCAGCAACGCGGCCTTTTTACGGTTCCTGGCCT
TTTGCTGGCCTTTTGCTCACATGTTCTTTCCTGCGTTATCCCCTGATTCTGTGGATAACCGTATTACCGCCTTTGAGTGAGCTGATACCGCTCGCCGCAGCCGAACGACCGAGCGCAGCGAGTC
AGTGAGCGAGGAAGCGGAAGAGCGCCTGATGCGGTATTTTCTCCTTACGCATCTGTGCGGTATTTCACACCGCATATGGTGCACTCTCAGTACAATCTGCTCTGATGCCGCATAGTTAAGCCA
GTATACACTCCGCTATCGCTACGTGACTGGGTCATGGCTGCGCCCCGACACCCGCCAACACCCGCTGACGCGCCCTGACGGGCTTGTCTGCTCCCGGCATCCGCTTACAGACAAGCTGTGACC
GTCTCCGGGAGCTGCATGTGTCAGAGGTTTTCACCGTCATCACCGAAACGCGCGAGGCAGCAGATCAATTCGCGCGCGAAGGCGAAGCGGCATGCATAATGTGCCTGTCAAATGGACGAAG
CAGGGATTCTGCAAACCCTATGCTACTCCGTCAAGCCGTCAATTGTCTGATTCGTTACCAATTATGACAACTTGACGGCTACATCATTCACTTTTTCTTCACAACCGGCACGGAACTCGCTCGGG
CTGGCCCCGGTGCATTTTTTAAATACCCGCGAGAAATAGAGTTGATCGTCAAAACCAACATTGCGACCGACGGTGGCGATAGGCATCCGGGTGGTGCTCAAAAGCAGCTTCGCCTGGCTGAT
ACGTTGGTCCTCGCGCCAGCTTAAGACGCTAATCCCTAACTGCTGGCGGAAAAGATGTGACAGACGCGACGGCGACAAGCAAACATGCTGTGCGACGCTGGCGAT

Supplementary Table S2. Reagents and antibodies used for flow cytometry.

Antigen	Clone	Fluorophore	Dilution Used for Staining	Source
Fixable Viability Dye		eFluor™ 506	1:800	Thermo Fisher Scientific
Cd45	30-F11	AlexaFluor700	1:400	Biolegend
Cd11b	M1/70	PE-Dazzle	1:800	Biolegend
Cd11c	N418	APC-Cy7	1:400	Biolegend
Cd19	1D3/CD19	PerCP-Cy5.5	1:200	Biolegend
Cd8	53-5.8	BrilliantViolet650	1:200	Biolegend
Cd4	RM4-5	BrilliantViolet570	1:400	Biolegend
F4/80	BM8	PE-Cy7	1:400	Biolegend

Supplementary Table S3. Size distribution and zeta-potentials of CPP/mRNA and CPP/pDNA nanoparticles formed at CPP/NA charge ratio 3:1.

	Size distribution				Zeta-potential	
CPP	mRNA, CR3		pDNA, CR3		mRNA, CR3	pDNA, CR3
	Size, nm	pDI	Size, nm	pDI		
PF14	166 ± 11	0.34 ± 0.03	188 ± 8	0.21 ± 0.01	46.4 ± 0.49	36.7 ± 0.36
NF71	165 ± 14	0.35 ± 0.06	134 ± 2	0.29 ± 0.02	37.0 ± 0.25	37.4 ± 0.63
NF55	186 ±10	0.24 ± 0.01	163 ± 2	0.30 ± 0.05	33.6 ± 0.01	38.2 ±1.01
NF430	167 ± 2	0.22 ± 0.01	143 ± 2	0.16 ± 0.01	35.5 ± 0.71	46.8 ± 1.45
NF424	172 ± 13	0.31 ± 0.02	138 ± 6	0.14 ± 0.03	34.6 ± 0.32	42.1± 1.13
NF436	177 ± 5	0.35 ± 0.02	140 ± 1	0.18 ± 0.01	33.8 ± 0.47	35.8 ± 0.65

Supplementary Figures:

MALDI and UPLC data of CPPs used in this work.

PF14

UPLC retention time: 12 min 50 s

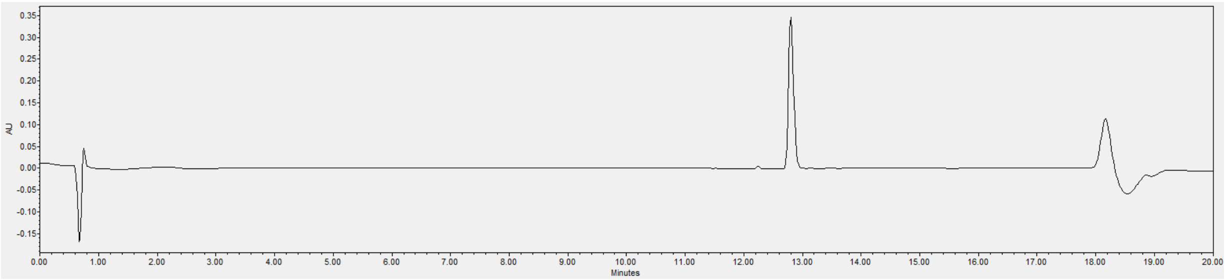

MALDI main peak 2405 m/z

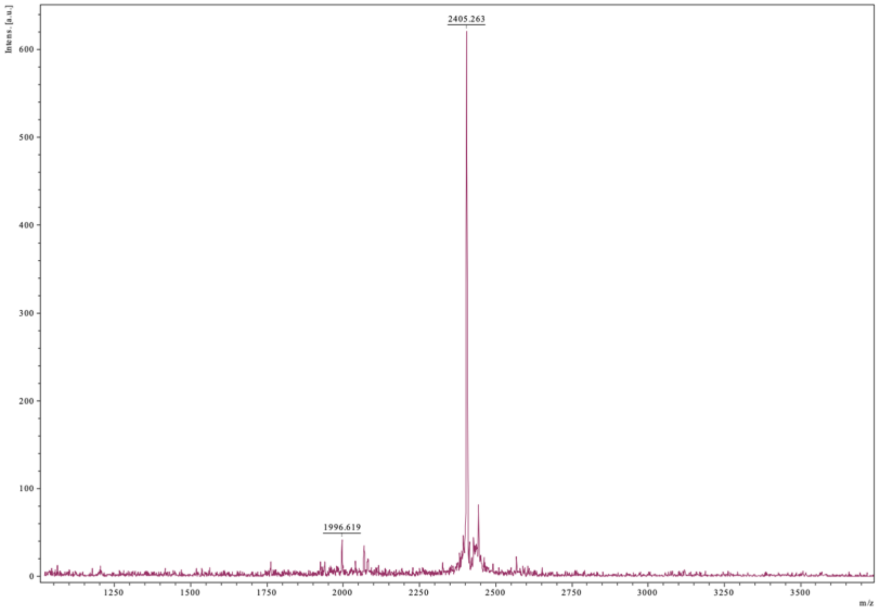

NF55

UPLC retention time 13 min 12 s

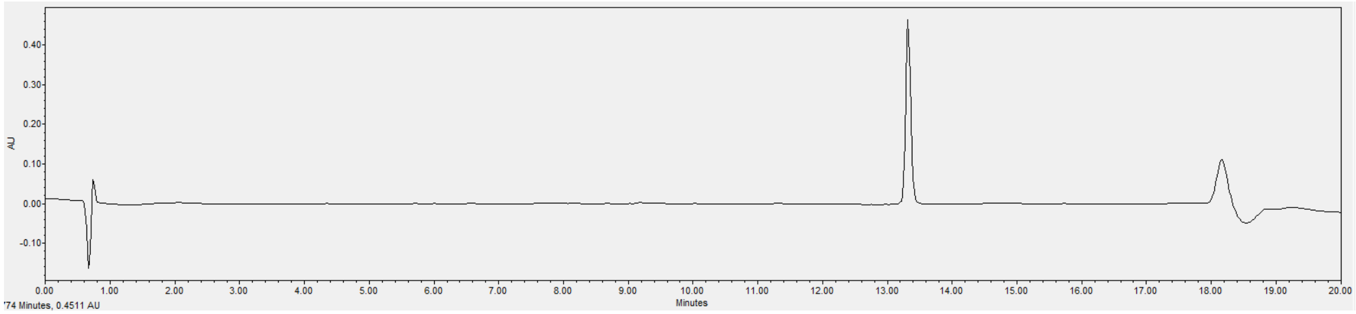

MALDI main peak 2376 m/z

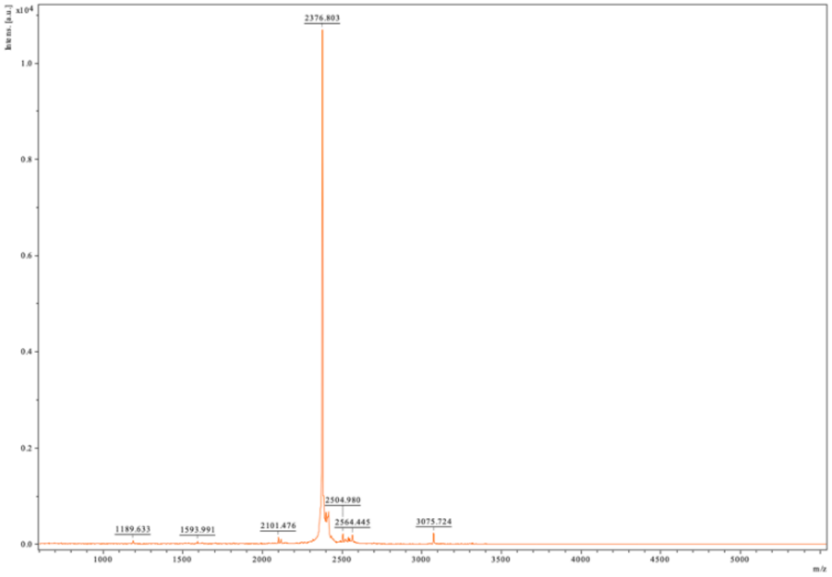

NF71

UPLC retention time 9 min 36 s

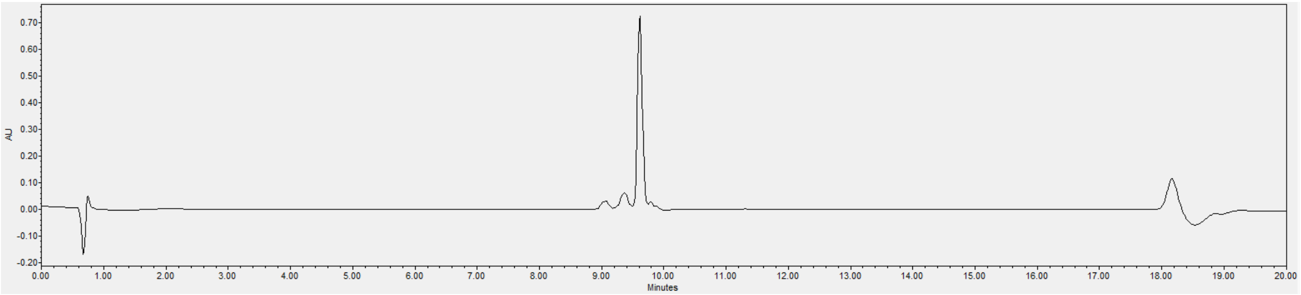

MALDI main peak 2627 m/z

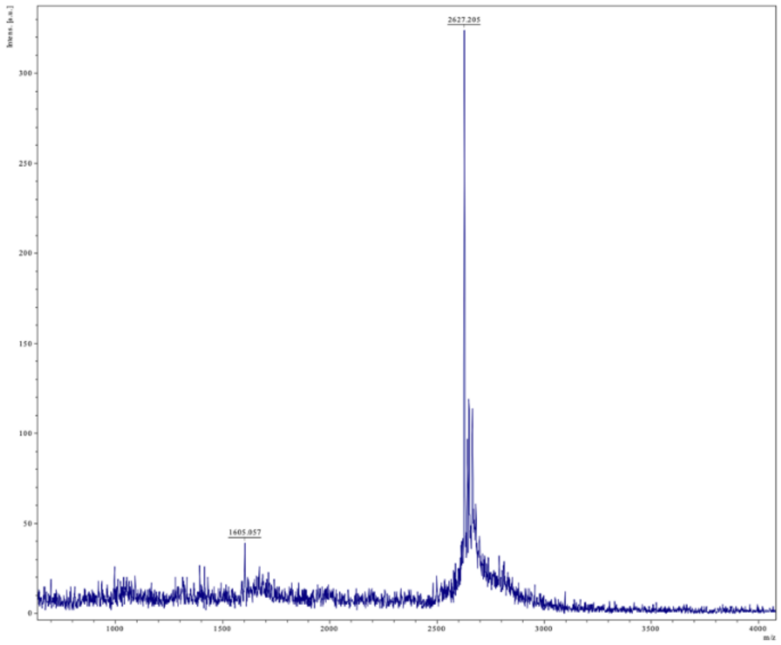

NF430

UPLC retention time 13 min 6 s

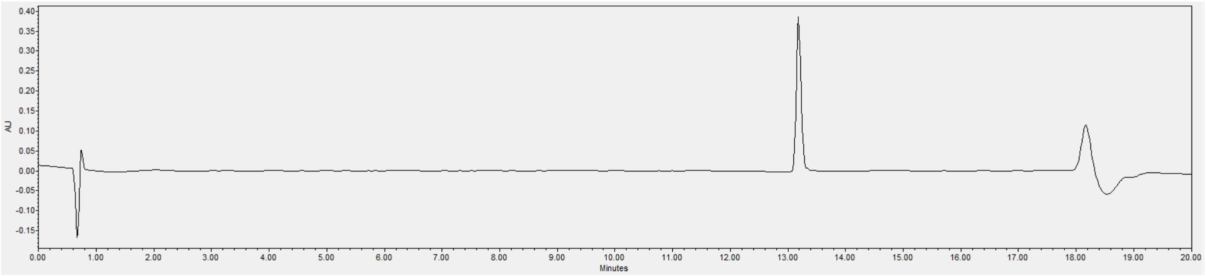

MALDI main peak 2149 m/z

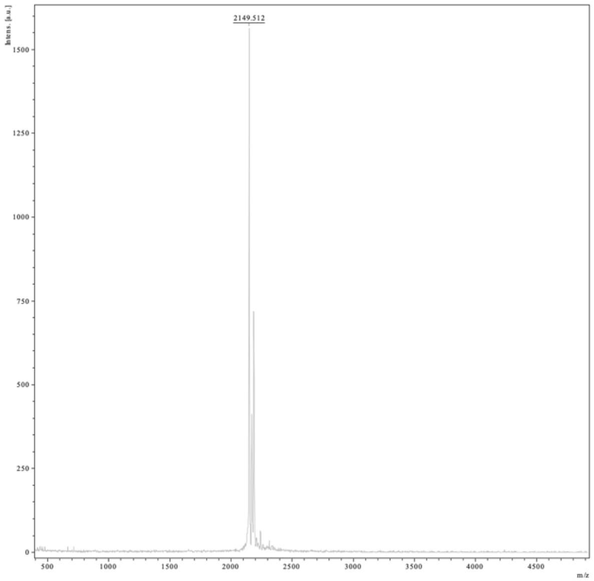

NF424

UPLC retention time 13 min

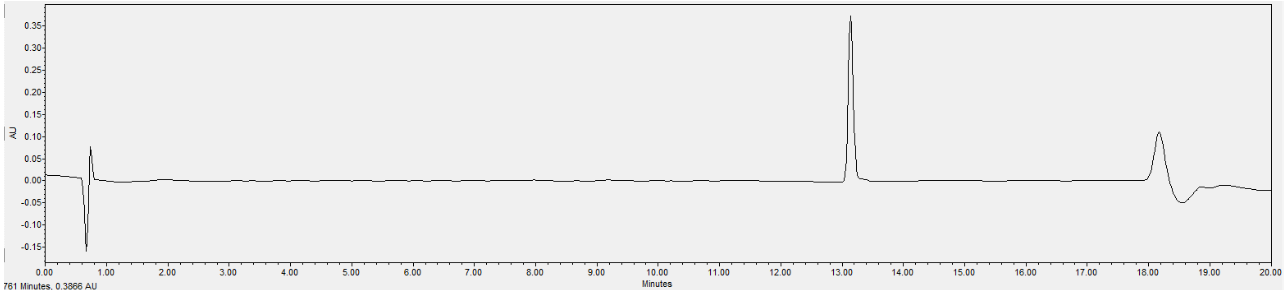

MALDI main peak 2136 m/z

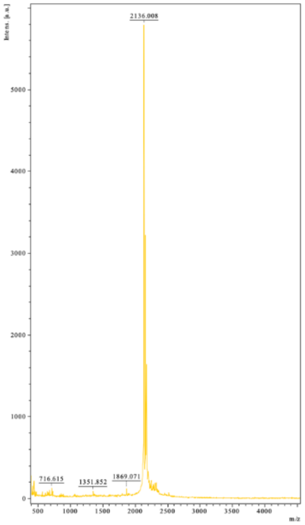

NF436

UPLC retention time 12 min 55 s

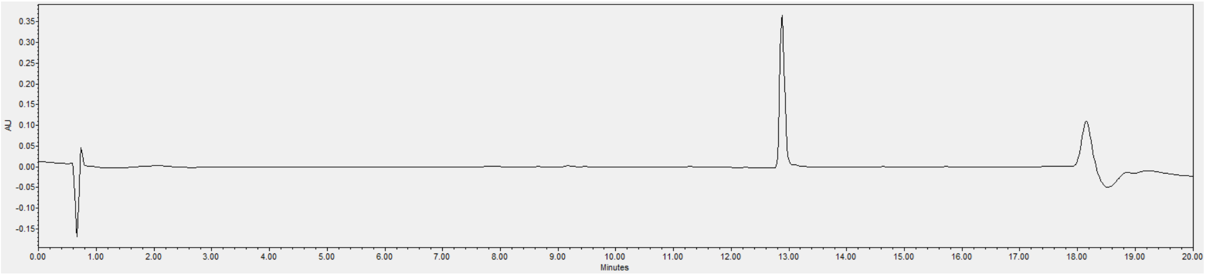

MALDI main peak 2137 m/z

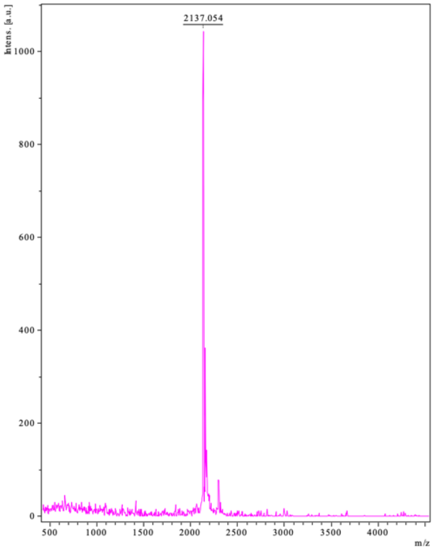

Figure S1. HPLC and MS for used peptides. Peptides were analyzed with reverse-phase ultra-performance liquid chromatography (ACQUITY UPLC H-class (Waters, USA)) accompanied with Empower software and equipped with C18 column (ACQUITY UPLC® BEH130 C18, 1.7 µm, 100 × 2.1 mm) used with temperature set to 40°C. The gradient was set from 5 – 95 % acetonitrile/water supplemented with 0.1% TFA, over a period of 13 min. The molecular weight of the peptides was determined by matrix-assisted laser desorption-ionization/time of flight mass spectrometry (Brucker Microflex LT/SH, USA).

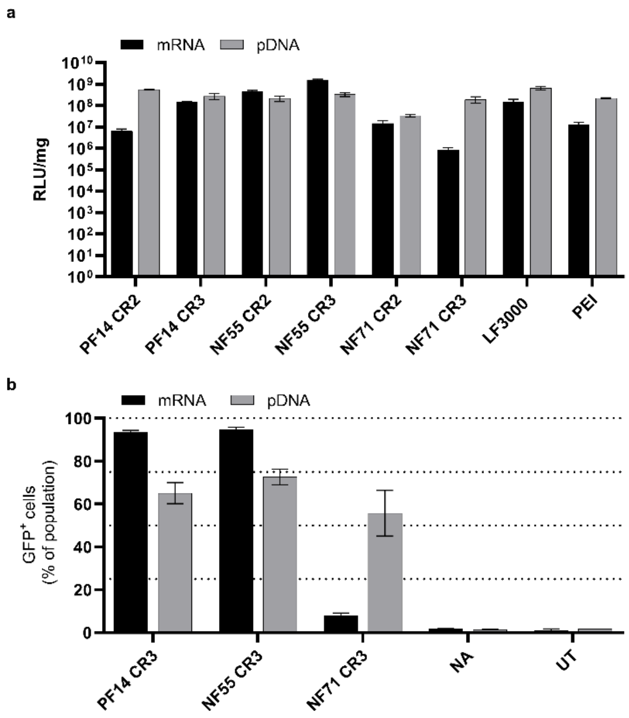

Figure S2. Total reporter levels and transfected cell population in vitro. All CPPs achieve high expression levels with both pDNA and mRNA in CHO-K1 cells. a) Transfection efficacy based on total reporter levels 24 h post-transfection from whole cell lysate. As commercially available transfection reagents Lipofectamine 3000 (LF3000) and PEI-Max (PEI) were used. PEI/NA complexes were formed at N/P ratio 20. Transfection with 0.1 µg of nucleic acid per 96-well plate well in 100 µl of serum (10% FBS) containing media. 24 h post-transfection cells were lysed, and from cell lysate relative luminescence (RLU) were detected after addition of substrate. RLU vales are normalized to total protein in the whole cell lysate b) transfected cell population percentage determined with flow cytometry. CHO-K1 cells were transfected with 0.5 µg of NA per 24-well plate well and in 500 µl of serum containing media. 4 h post-transfection media was replaced with fresh media and cells were further incubated for 20 h. 24 h post-transfection cells were detached and analyzed with flow cytometer to detect fluorescent positive cell population (GFP+) out of live cells. For threshold setting untreated cells were used.

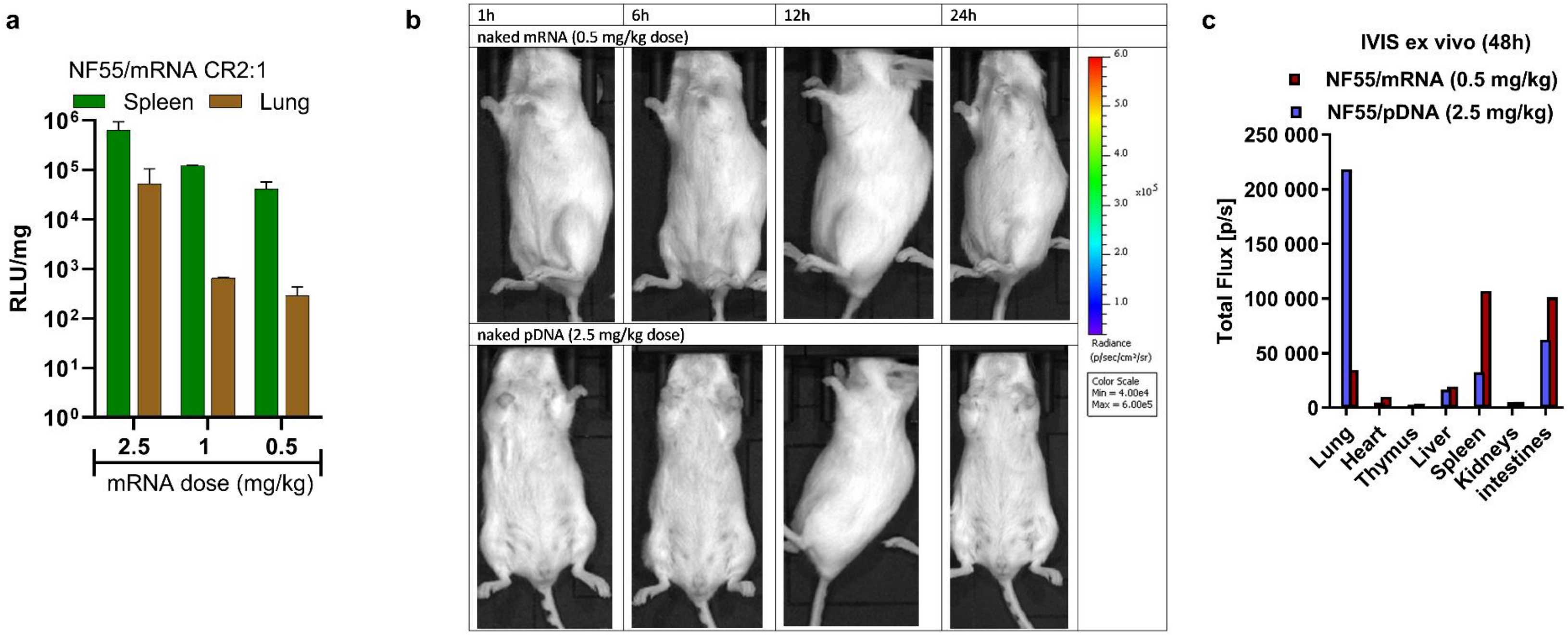

Figure S3. In vivo screening of NF55/mRNA and controls groups for imaging. mRNA dose reduction in NF55/mRNA complexes results in reduction of expression in the lungs whereas in the spleen the reporter levels remain relatively high even with the lower dose. Tissues were harvested 24 h post-injection of NF55/mRNA complexes. 2.5 mg/kg, 1 mg/kg and 0.5 mg/kg doses of mRNA in the complex were compared. b) Representative images from whole body imaging with equalized radiance scales shown in Figure 1e. Mice treated with 0.5 mg/kg of luciferase encoding mRNA or 2.5 mg/kg luciferase encoding pDNA. Images taken at 1h, 6h, 12h and 24h post-administration. c) Ex vivo imaging of tissues collected 48 h post injection.

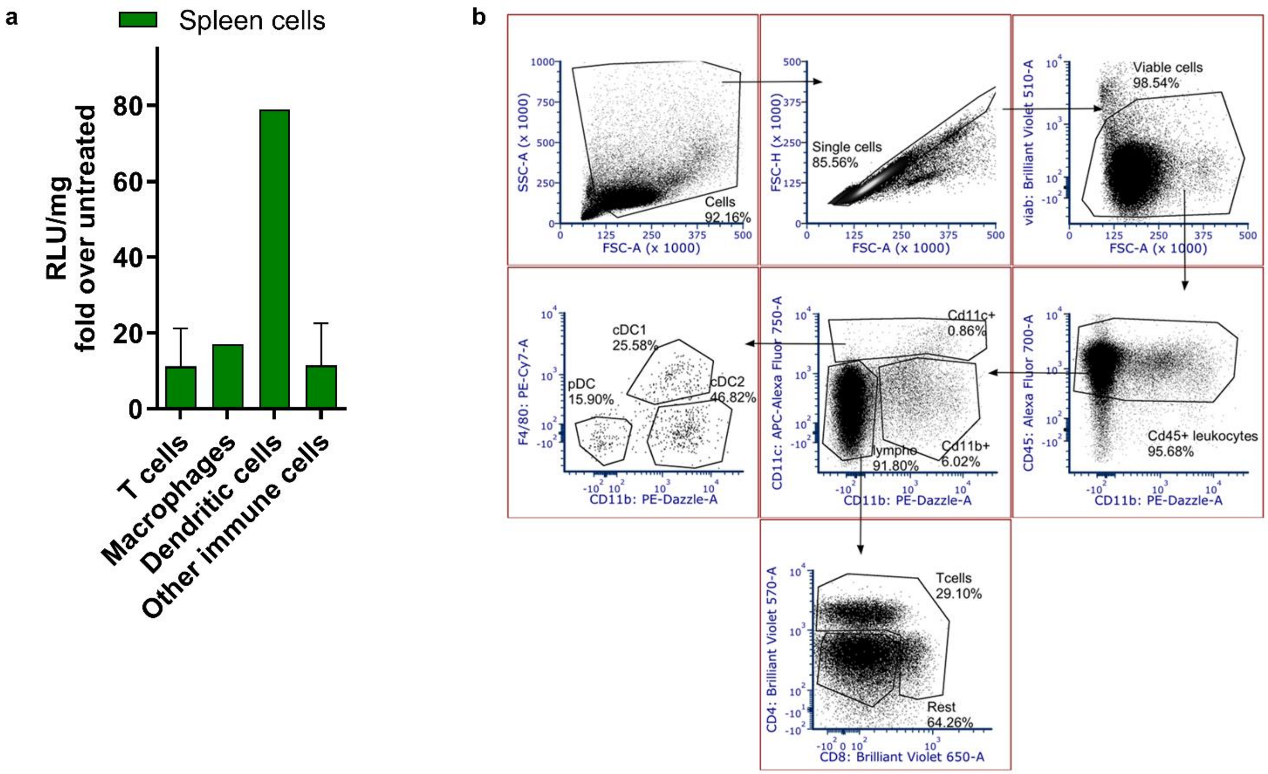

Figure S4. Gating strategy for sorting cells from spleen tissue from NF55/mRNA treated mice and reporter levels detected from different steps of sorting and from sorted cells. Spleen harvested 24 h post-administration of complexes via single i.v. injection. a) Detected reporter levels after single administration of NF55/mRNA. Samles were collected and lysed to detect expressed luciferase levels in the sorted cell lysate.

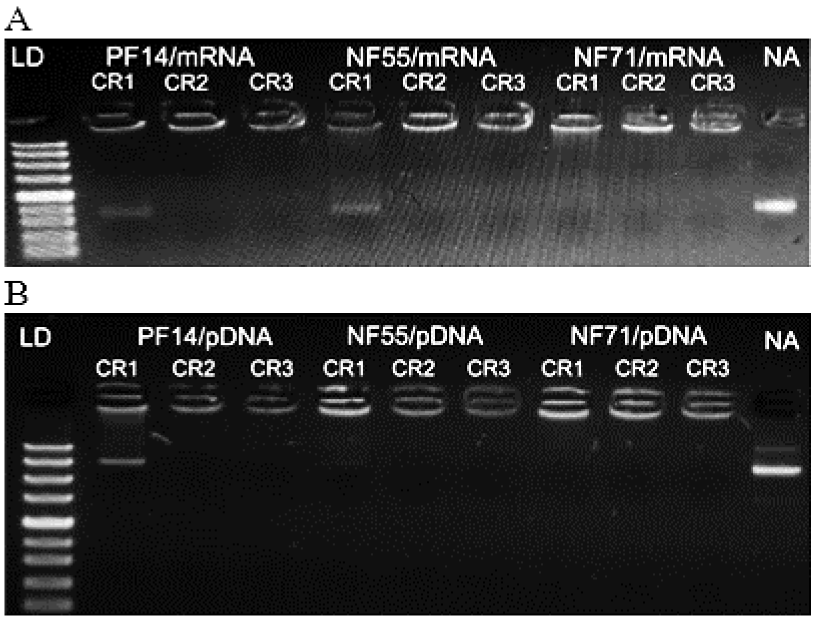

Ladder ZipRuler2:

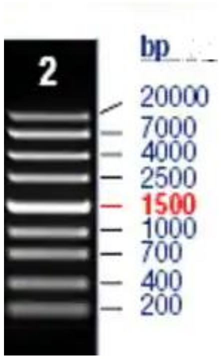

Figure S5. A) CPPs are efficiently able to interact with mRNA and limit its migration in 1% agarose gel. CPP/mRNA complexes were formed at charge ratios (CR) 1:1; 2:1 and 3:1 with CPP in excess. After incubation samples were diluted, loading dye was added and samples transferred to agarose gel tooth. LD – ladder ZipRuler 2. NA – free mRNA at the same concentration as used in the CPP/NA complexes. B) CPPs are efficiently able to interact with pDNA and limit its migration in agarose gel. CPP/pDNA complexes were formed at charge ratios (CR) 1:1; 2:1 and 3:1 with CPP in excess. After incubation samples were diluted, loading dye was added and samples transferred to agarose gel tooth. LD – ladder ZipRuler 2. NA – free pDNA at the same concentration as used in the CPP/NA complexes.

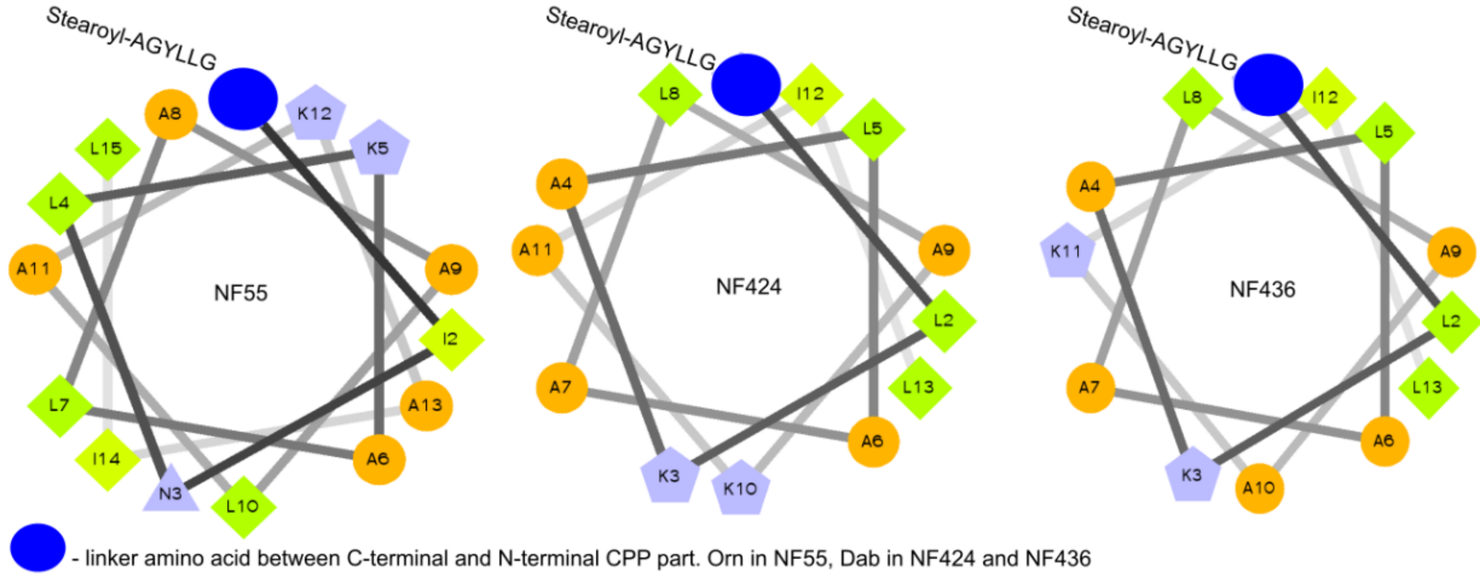

Figure S6. Helical wheel projections of NF55, NF424, NF436 C-terminal pert from the linker amino acid. Projections are modified from the calculations done in: http://rzlab.ucr.edu/scripts/wheel/wheel.cgi.

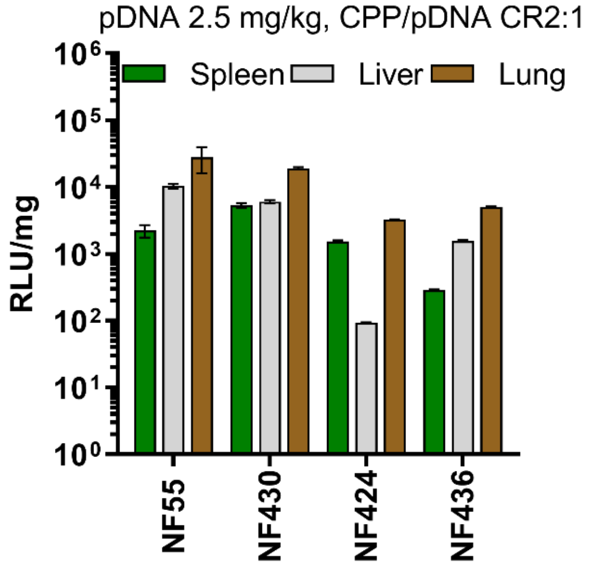

Figure S7. The in vivo efficacy of CPP/pDNA complexes formed with new CPPs. CPP/pDNA complexes were formed at charge ratio (CR) 2:1 with CPP in excess. The reporter levels from delivered luciferase encoding pDNA was detected from tissue homogenate 16 h post-injection.

Statistical Analysis

Fig1A

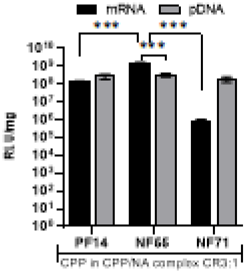

2-way ANOVA

Effect	Univariate Tests of Significance, Effect Sizes, and Powers for Transfection in Cells (Fig1A statistics)<br>Sigma-restricted parameterization<br>Effective hypothesis decomposition					
	SS	Degr. of Freedom	MS	F	p	Partial eta-squared
Intercept	1.750005E+19	1	1.750005E+19	38.61421	0.000000	0.233158
CPP	1.870530E+19	2	9.352649E+18	20.63681	0.000000	0.245277
Nucleic Acid	2.214110E+18	1	2.214110E+18	4.88548	0.028875	0.037043
CPP*Nucleic Acid	1.431876E+19	2	7.159382E+18	15.79732	0.000001	0.199216
Error	5.755669E+19	127	4.532023E+17			

Post-hoc Tukey tests

Cell No.	Tukey HSD test; variable Transfection in Cells (Fig1A)<br>Approximate Probabilities for Post Hoc Tests<br>Error: Between MS = 453E15, df = 127.00							
	CPP	Nucleic Acid	{1}<br>1427E5	{2}<br>2766E5	{3}<br>1542E6	{4}<br>3317E5	{5}<br>8491E2	{6}<br>1929E5
1	PF14	mRNA		0.994908	0.000020	0.972206	0.997128	0.999967
2	PF14	pDNA	0.994908		0.000020	0.999651	0.883714	0.998653
3	NF55	mRNA	0.000020	0.000020		0.000020	0.000020	0.000020
4	NF55	pDNA	0.972206	0.999651	0.000020		0.756018	0.982465
5	NF71	mRNA	0.997128	0.883714	0.000020	0.756018		0.979161
6	NF71	pDNA	0.999967	0.998653	0.000020	0.982465	0.979161	

Fig1B

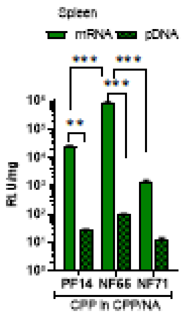

2-way ANOVA

Effect	Univariate Tests of Significance, Effect Sizes, and Powers for Expression in Spleen (Fig1B)<br>Sigma-restricted parameterization<br>Effective hypothesis decomposition<br>SS	Degr. of Freedom	MS	F	p	Partial eta-squared
Intercept	2.834192E+11	1	2.834192E+11	8843 .910	0 .00	0 .998194
CPP	7.887965E+11	3	2.629322E+11	8204 .626	0 .00	0 .999350
Nucleic Acid	2.832424E+11	1	2.832424E+11	8838 .393	0 .00	0 .998193
CPP*Nucleic Acid	7.884898E+11	3	2.628299E+11	8201 .435	0 .00	0 .999350
Error	5.127491E+08	16	3.204682E+07			

Post-hoc Tukey tests

Tukey HSD test; variable Expression in Spleen (Fig1B)
Approximate Probabilities for Post Hoc Tests
Error: Between MS = 3205E4, df = 16.000

Cell No.	CPP	Nucleic Acid	{1}<br>24224.	{2}<br>27.210	{3}<br>8963E2	{4}<br>100.40	{5}<br>1393.6	{6}<br>11.659	{7}<br>11.221	{8}<br>4.5525
1	PF14	mRNA		0 .003	0 .000	0 .003	0 .017	0 .003	0 .011	0 .003
2	PF14	pDNA	0 .003		0 .000	1 .000	1 .000	1 .000	1 .000	1 .000
3	NF55	mRNA	0 .000	0 .000		0 .000	0 .000	0 .000	0 .000	0 .000
4	NF55	pDNA	0 .003	1 .000	0 .000		1 .000	1 .000	1 .000	1 .000
5	NF71	mRNA	0 .017	1 .000	0 .000	1 .000		1 .000	1 .000	1 .000
6	NF71	pDNA	0 .003	1 .000	0 .000	1 .000	1 .000		1 .000	1 .000
7	NA	mRNA	0 .011	1 .000	0 .000	1 .000	1 .000	1 .000		1 .000
8	NA	pDNA	0 .003	1 .000	0 .000	1 .000	1 .000	1 .000	1 .000	

Fig2E

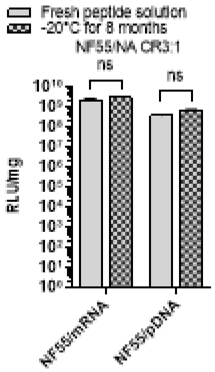

2-way ANOVA

Effect	Univariate Tests of Significance for Transfection in Cells (Fig2E)<br>Sigma-restricted parameterization<br>Effective hypothesis decomposition<br>SS	Degr. of Freedom	MS	F	p
Intercept	9.999625E+19	1	9.999625E+19	144.1409	0.000000
CPP storage	3.581624E+18	1	3.581624E+18	5.1628	0.029328
Nucleic acid	4.414400E+19	1	4.414400E+19	63.6319	0.000000
CPP storage*Nucleic acid	8.377574E+17	1	8.377574E+17	1.2076	0.279311
Error	2.428089E+19	35	6.937398E+17		

Post-hoc Tukey tests

Effect	Univariate Tests of Significance for Transfection in Cells (Fig2E)<br>Sigma-restricted parameterization<br>Effective hypothesis decomposition<br>SS	Degr. of Freedom	MS	F	p
Intercept	9.999625E+19	1	9.999625E+19	144.1409	0.000000
CPP storage	3.581624E+18	1	3.581624E+18	5.1628	0.029328
Nucleic acid	4.414400E+19	1	4.414400E+19	63.6319	0.000000
CPP storage*Nucleic acid	8.377574E+17	1	8.377574E+17	1.2076	0.279311
Error	2.428089E+19	35	6.937398E+17		

Fig2F

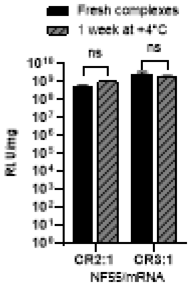

2-way ANOVA

Effect	Univariate Tests of Significance for Transfection in Cells (Fig2F)<br>Sigma-restricted parameterization<br>Effective hypothesis decomposition<br>SS	Degr. of Freedom	MS	F	p
Intercept	4.559942E+19	1	4.559942E+19	39.55488	0.000011
CPP Storage	1.440317E+17	1	1.440317E+17	0.12494	0.728352
CR	1.059106E+19	1	1.059106E+19	9.18713	0.007948
CPP Storage*CR	2.388614E+18	1	2.388614E+18	2.07199	0.169304
Error	1.844503E+19	16	1.152814E+18		

Post-hoc Tukey tests

Cell No.	Tukey HSD test; variable Transfection in Cells (Fig2F)<br>Approximate Probabilities for Post Hoc Tests<br>Error: Between MS = 115E16, df = 16.000<br>CPP Storage	CR	{1}<br>5215E5	{2}<br>2668E6	{3}<br>1043E6	{4}<br>1807E6
1	Fresh complex	CR2		0.027950	0.867748	0.269623
2	Fresh complex	CR3	0.027950		0.118742	0.595264
3	1 week at +4°C	CR2	0.867748	0.118742		0.679818
4	1 week at +4°C	CR3	0.269623	0.595264	0.679818	

Fig2G

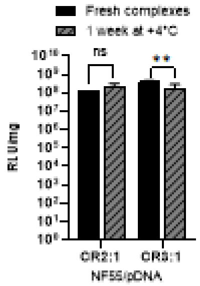

2-way ANOVA

Effect	Univariate Tests of Significance for Transfection in Cells (Fig2G)<br>Sigma-restricted parameterization<br>Effective hypothesis decomposition<br>SS	Degr. of Freedom	MS	F	p
Intercept	1.011827E+18	1	1.011827E+18	89.17609	0.000000
CPP Storage	4.218565E+16	1	4.218565E+16	3.71798	0.071769
CR	7.786244E+16	1	7.786244E+16	6.86231	0.018585
CPP Storage*CR	1.627730E+17	1	1.627730E+17	14.34580	0.001614
Error	1.815422E+17	16	1.134639E+16		

Post-hoc Tukey tests

Cell No.	Tukey HSD test; variable Transfection in Cells (Fig2G)<br>Approximate Probabilities for Post Hoc Tests<br>Error: Between MS = 113E14, df = 16.000<br>CPP Storage	CR	{1}<br>1182E5	{2}<br>4235E5	{3}<br>2068E5	{4}<br>1512E5
1	Fresh complex	CR2		0.001873	0.567318	0.960513
2	Fresh complex	CR3	0.001873		0.025087	0.004827
3	1 week at +4°C	CR2	0.567318	0.025087		0.841493
4	1 week at +4°C	CR3	0.960513	0.004827	0.841493	

Fig4B

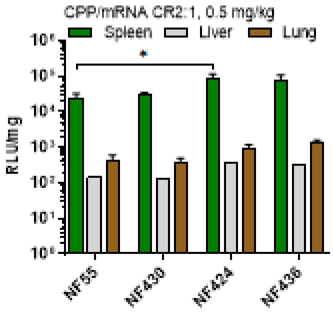

2-way ANOVA

Effect	Univariate Tests of Significance for Expression (Fig4B)<br>Sigma-restricted parameterization<br>Effective hypothesis decomposition<br>SS	Degr. of Freedom	MS	F	p
Intercept	2.422164E+10	1	2.422164E+10	22.46248	0.000014
CPP	6.802097E+09	3	2.267366E+09	2.10269	0.109334
Tissue	4.585485E+10	2	2.292743E+10	21.26226	0.000000
CPP*Tissue	1.303258E+10	6	2.172097E+09	2.01434	0.077635
Error	6.469893E+10	60	1.078316E+09		

Post-hoc Tukey tests

Tukey HSD test; variable Expression (Fig4B)
Approximate Probabilities for Post Hoc Tests
Error: Between MS = 1078E6, df = 60.000

Cell No.	CPP	Tissue	{1} 147.37	{2} 24922.	{3} 442.24	{4} 127.63	{5} 31677.	{6} 397.00	{7} 394.06	{8} 91564.	{9} 968.67	{10} 323.33	{11} 74608.	{12} 1301.0
1	NF55	Liver		0.975	1.000	1.000	0.938	1.000	1.000	0.000	1.000	1.000	0.011	1.000
2	NF55	Spleen	0.975		0.977	0.989	1.000	0.990	0.962	0.018	0.968	0.976	0.292	0.983
3	NF55	Lung	1.000	0.977		1.000	0.942	1.000	1.000	0.000	1.000	1.000	0.012	1.000
4	NF430	Liver	1.000	0.989	1.000		0.967	1.000	1.000	0.002	1.000	1.000	0.037	1.000
5	NF430	Spleen	0.938	1.000	0.942	0.967		0.969	0.918	0.141	0.927	0.941	0.675	0.952
6	NF430	Lung	1.000	0.990	1.000	1.000	0.969		1.000	0.002	1.000	1.000	0.038	1.000
7	NF424	Liver	1.000	0.962	1.000	1.000	0.918	1.000		0.000	1.000	1.000	0.005	1.000
8	NF424	Spleen	0.000	0.018	0.000	0.002	0.141	0.002	0.000		0.000	0.000	0.998	0.000
9	NF424	Lung	1.000	0.968	1.000	1.000	0.927	1.000	1.000	0.000		1.000	0.006	1.000
10	NF436	Liver	1.000	0.976	1.000	1.000	0.941	1.000	1.000	0.000	1.000		0.011	1.000
11	NF436	Spleen	0.011	0.292	0.012	0.037	0.675	0.038	0.005	0.998	0.006	0.011		0.013
12	NF436	Lung	1.000	0.983	1.000	1.000	0.952	1.000	1.000	0.000	1.000	1.000	0.013	
